# Per- and Polyfluoroalkyl (PFAS) Disruption of Thyroid Hormone Synthesis

**DOI:** 10.1021/acsomega.4c03578

**Published:** 2024-09-10

**Authors:** Semiha Kevser Bali, Rebecca Martin, Nuno M. S. Almeida, Catherine Saunders, Angela K. Wilson

**Affiliations:** Department of Chemistry and MSU Center for PFAS Research, Michigan State University, East Lansing, Michigan 48864, United States

## Abstract

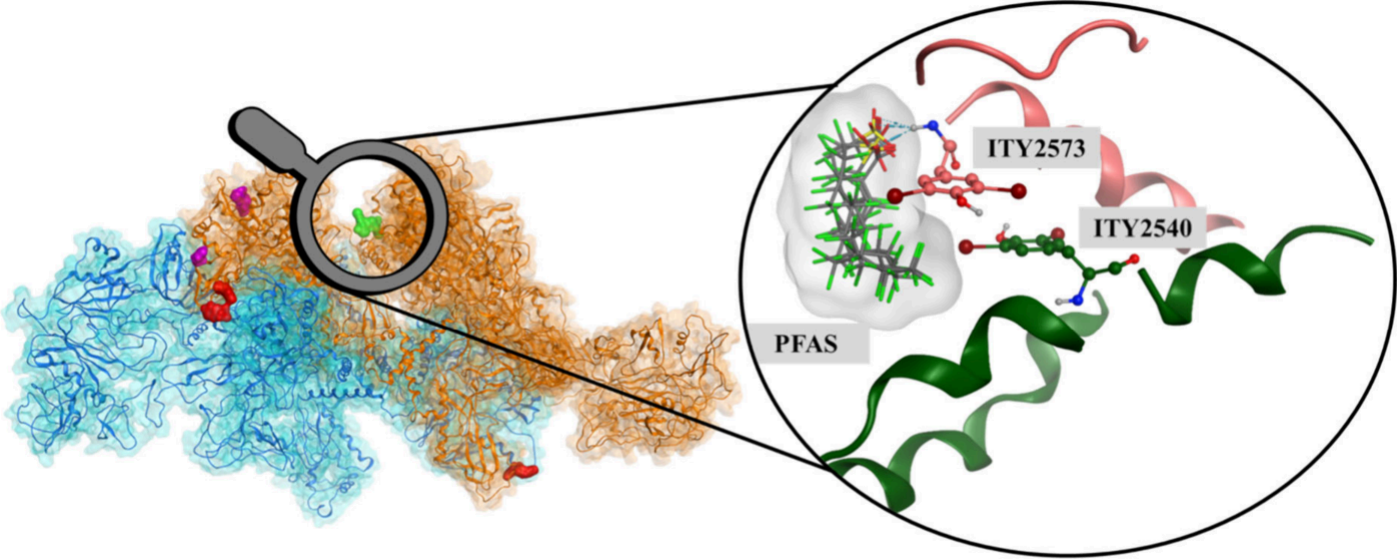

Per- and polyfluoroalkyl substances (PFAS) are a group of environmental pollutants that have been linked to a variety of health problems in humans, including the disruption of thyroid functions. Herein, for the first time, the impact of PFAS on thyroid hormone synthesis is shown. Mid- to long-chain PFAS impact thyroid hormone synthesis by changing the local hydrogen bond network as well as the required orientation of hormonogenic residues, stopping the production of thyroxine (T4). Furthermore, the toxic effects of sulfonic PFAS are more prominent than those of carboxylic PFAS, highlighting that the exposure to these specific compounds can pose greater problems for thyroid homeostasis.

## Introduction

Environmental pollutants can significantly impact the health of living organisms in the ecosystem and in human populations. Some of the most recent health concerns related to environmental pollutants have been attributed to per- and polyfluoroalkyl substances (PFAS), a group of man-made chemicals with broad industrial applications due to their unmatched water- and oil-repellent properties as well as heat-resistance.^1−3^ There are more than ∼14,000 compounds listed in the EPA PFASTRUCT database as of June 2023; however, remarkably, only approximately one percent of them have been tested for their toxicities.^4,5^ PFAS can be found in many products with nonstick and water-repellent surfaces, including food packaging, water-resistant clothing and shoes, and firefighting foams, to provide only a small number of examples, and are often referred to as “forever chemicals” or “zombie chemicals” due to their resistance to degradation. The resistance to degradation, consequently, has resulted in bioaccumulation of PFAS compounds in humans and animals, which has been linked to disruptions of glucose and bile acid metabolisms, immune, reproductive, and thyroid systems, and lipid homeostasis.^6−13^

**Figure 1:**
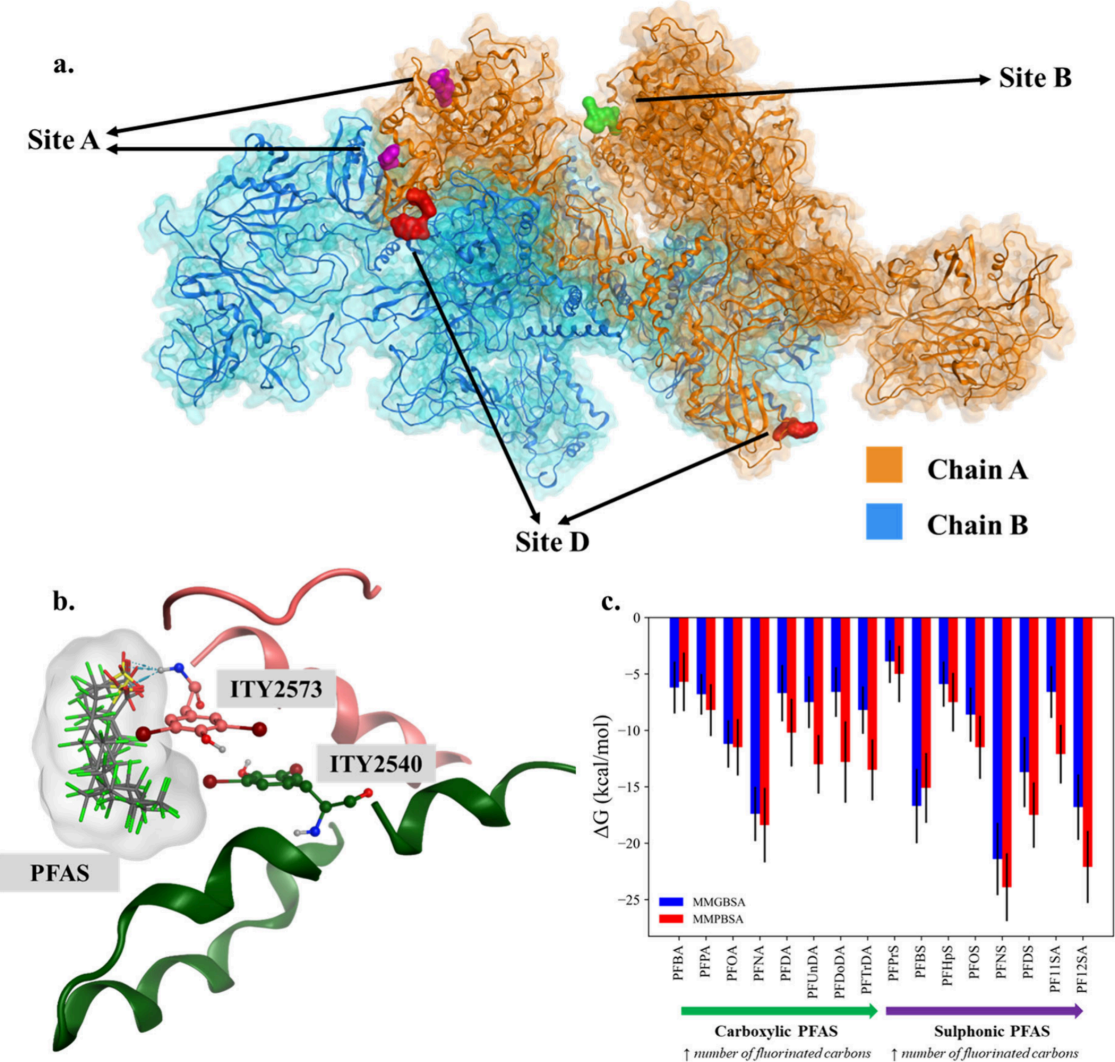
(a) The dimeric structure of human thyroglobulin (hTG) and the three hormonogenic sites on Chain A are shown. Among the identified hormogenic sites, Site A has two potential donor residues, and Site D has hormonogenic tyrosines from both chains. (b) The docking poses for PFAS in Site B along with ITY residues. (c) The binding energies for investigated PFAS, calculated with MM-GBSA and MM-PBSA methods, are shown along with the standard deviations. Carboxylic and sulfonic PFAS are included.

To provide a backdrop for the potential impact of PFAS on thyroid systems, a functional thyroid gland is crucial for neurodevelopment, cognitive and behavioral growth, as well as regulation of the metabolic rate.^11^ The synthesis of thyroid hormones thyroxine (T4) and triiodothyronine (T3) is performed by thyroglobulin, which is a highly conserved protein in vertebrates, and thyroglobulin is located in the lumen of the thyroid follicles.^14^ In humans, the thyroglobulin protein—called the human thyroglobulin (hTG) protein—is a homodimer and has four hormonogenic sites (sites A to D as shown in Figure 1), the four sites where the T4 hormone is produced.^15−17^ These sites on hTG are the locations where thyroid hormones are synthesized. Although the exact mechanism is still not fully understood, the available cryo-EM structures indicate that the orientation of ITY residues as well as neighboring lysine and phenylalanine residues are crucial for the mechanism to take place.^15,17^

Current research on the impact of PFAS on thyroid function is mainly based on epidemiological studies and clinical data, with mixed conclusions as to whether PFAS leads to an increase or decrease in thyroid hormone levels. One study in which the associations between PFAS exposure during pregnancy and the neurodevelopment in infants were investigated indicated a relationship with PFHxS and PFBS exposure, linking to thyroid hormone-mediated neurodevelopment problems.^12^ Prior studies have shown that during pregnancy, there is an association between the maternal levels of thyroid stimulating hormone and the PFHxS, PFNA, and PFOA concentrations.^18−23^ Animal studies in rats indicated a decrease in T3 and T4 levels upon PFOA and PFOS exposure,^24,25^ while long-term exposure to PFNA was linked to an increase in T3 levels in zebrafish.^25,26^

While there is no single mechanism in which PFAS could disrupt the thyroid system, there are *in silico* and *in vitro* studies addressing various potential targets.^27^ One study investigated the sodium-iodide symporters for rat and human thyroid cell lines and found that PFOS and PFHxS inhibited this protein.^27,28^ In a number of prior studies, PFAS exposure was proposed to alter the expression of proteins important for iodide removal and thyroid hormone signaling.^24,29−31^ A study of PFAS’ effects on the thyroid was performed on common carp fish,^32^ and Manera et al. suggested that the PFOA concentration can cause significant effects on the thyroid follicles of carp by disrupting production as well as reabsorption of thyroglobulin.

As the PFAS toxicity on thyroid chemistry is a complicated and mainly uncharted process, the source of the thyroid hormone production, namely, hTG, and the influence of PFAS on the thyroid hormone synthesis have been investigated. Understanding how PFAS can impact homeostasis in humans will provide insight toward the development of potential mitigation strategies, such as targeted treatments and interventions for thyroid-related health issues.

## Results

### PFAS Binding

The location of Sites A, B, and D, and the docking poses of PFAS are shown. All of the functional groups that point toward the selected PFAS ITY2573 residue are shown in Figure 1(a). Site B of the hTG protein was selected for the suitability of the initial positioning of tyrosine residues, as Site A and Site D either have two donor ITY residues or have tyrosine residues from different chains. As the hTG monomer is a large protein with ∼2,700 residues, the root-mean-square deviations (RMSDs) and total energies were calculated for the whole simulation length to assess if the simulated systems converged structurally and energetically (Tables S2–S3 for RMSD, Tables S4–S5 for total energies). For the majority of the hTG simulations, both the total energy and the RMSD values reached a plateau within the last 5 ns of the simulations (as reported in the SI); hence, the last 5 ns of the simulations were considered for analysis. Both the PFAS and the binding site showed no significant conformation change during this simulation period.

The binding energies for each hTG/PFAS complex were calculated using end-point methods (MM-GBSA/PBSA) to estimate the relative binding strength of carboxylic and sulfonic PFAS with various fluorinated carbon chain lengths, as per Figure 1(c). Current literature indicates that the thyroid hormone synthesis in hTG can be affected negatively by the exposure to PFAS with eight to nine fluorinated carbons.^18−23,32^ In our simulations, carboxylic PFAS showed an increase in binding strengths as the fluorinated carbons increased from PFBA to PFNA. However, this observation was different for PFCA with more than nine carbons. For PFDA, PFUnDA, PFDoDA, and PFTrDA, the binding energies were in the range of −10 and −13 kcal/mol (MM-PBSA). The binding energy analyses of PFSA are quite different than for PFCA. Among the investigated sulfonic PFAS, the strongest binding energy was observed for PFNS. Interestingly, PFBS was also among the strong binders, which was previously noted by Yao et al.^12^ The binding strengths of longer-chain PFSA were also higher than those of their PFCA counterparts with the same fluorinated carbon chain length. These differences in binding strengths indicate that PFCA and PFSA compounds have different impacts on the binding site and, consequently, on the thyroid hormone synthesis.

Residue decomposition can help understand some of the energetic differences observed in Figure 1a), so they were calculated for each simulation of PFCA and PFSA compounds with the binding pocket residues, as shown in Table 1, and Tables S6–S9. The pocket residues are separated into four groups based on their polarity and acidity: polar, nonpolar, basic, and acidic residues. The strengths of the electrostatic interactions and van der Waals interactions made by PFAS and residues within 10 Å radius suggest that the basic residues showed the strongest interaction among all; specifically, K2536 had the highest interaction energy with all of the PFAS. The acidic residues mainly had weak and nonstabilizing interactions, with values larger than zero. In general, electrostatic interactions with charged residues had stronger interactions with PFCA, while PFSA molecules interacted with polar and nonpolar residues in the binding site through the C–F tail. As PFSA showed slightly higher MM-PBSA energies, and this suggests that the tail group of PFAS provides stronger anchoring to the surrounding residues than the head groups of PFAS.

**Table 1:** Sum of Per-Residue Decomposition Energies for Charged Residues and Polar and Nonpolar Residues (in kcal mol^–1^)

	PFBA	PFPA	PFOA	PFNA	PFDA	PFUnDA	PFDoDA	PFTrDA
Charged Res.	–49.45	–26.79	–20.23	–46.05	–33.59	–27.55	–60.72	–2.16
Polar and Nonpolar Res.	–14.05	–27.67	–32.90	–44.80	–33.93	–33.88	–42.54	–34.51
Sum	–63.50	–54.46	–53.13	–90.84	–67.52	–61.43	–103.26	–36.68

	PFPrS	PFBS	PFHpS	PFOS	PFNS	PFDS	PF11SA	PF12SA
Charged Res.	–29.38	–7.53	–29.40	–11.94	14.92	–19.83	–30.31	–32.76
Polar and Nonpolar Res.	–13.13	–57.99	–33.03	–44.97	–75.64	–50.17	–32.79	–49.90
Sum	–42.51	–65.52	–62.43	–56.90	–60.72	–70.00	–63.10	–82.65

The contributions from the ITY residues were also identified as they play a pivotal role in thyroid hormone synthesis. PFAS primarily formed stabilizing interactions with ITY residues, although these interactions were weaker than those with charged residues. Among the PFAS with highest ITY interaction energies, PFNA, PFNS, and PF12SA exhibited high MM-PBSA energies, suggesting that ITY interactions could be the determining factor in predicting PFAS binding to Site B.

The interactions between the PFAS and ITY were established between the diiodotyrosine side chains and the C–F tails (Figure S7), and the total contribution from ITY residues increased as the fluorinated carbon chain length increased in PFCA molecules, with the exceptions of PFNA and PFTrDA. This finding provides further evidence supporting the crucial role of the C–F tail in stabilizing PFAS binding.

The hydrogen bond interactions formed by PFAS during the simulations were also investigated and are reported in Table S10. PFAS with 8 to 10 carbons predominantly formed direct hydrogen bonds, and as the chain length increased or decreased, the number of hydrogen bonds formed by PFAS with the protein decreased. During the simulations, PFDS exhibited the highest number of interactions with pocket residues, followed by PFNS and PFBS. All three compounds formed hydrogen bonds with S430, Q431, and ITY2573 residues, which also had high interaction energies with PFDS, PFNS, and PFBS. Among the PFCA compounds, the highest number of interactions were observed for PFNA. These observations further support the notion that for PFAS with certain carbon chain lengths, the local interactions made with the headgroup and, more importantly, through the C–F tail are important determinants of being stronger binders, as compared to other PFAS. Furthermore, the strong interactions between the C–F tail of PFSA compounds and polar/nonpolar residues resulted in the sulfonic PFAS having higher binding energies than PFCA.

### Changes in Local Interaction Patterns upon PFAS Binding

To understand how the presence of PFAS in the selected positions of the thyroglobulin protein changes structural interactions, the residues located nearby PFAS were divided into three regions: Regions 1, 2, and 3 (Figure S4). Region 1 has a loop secondary structure; Regions 2 and 3 have α helix structures, and the calculated hydrogen bond percentages are reported in Tables S11–S13. In Region 1, the interactions observed in the presence of PFAS were not significantly different— the apo system has interactions that were not observed when PFAS was bound. For this region, the interactions did not show a distinct pattern either for headgroup type or the carbon chain length.

On the other hand, the interactions within Region 2, clearly showed a noteworthy pattern: as the fluorinated carbon chain length increased, the number of interactions observed within the binding site increased (Table S12). The highest interaction percentage in the apo simulations was for the S2534/A2538 residue pair, which is part of the α helix in Region 2, with the interaction occurring through their backbone atoms. S2534/A2538 interactions persisted in the majority of PFAS simulations, with the exception of the PFOA, PFNA, PFOS, and PFDA simulations. The rest of the dominant interactions in the apo system persisted for 25 to 35% of the simulation and were observed in the majority of PFAS simulations as well. The ITY2540/K2536 interaction persisted in ∼25% of the simulations in the apo system, and the interaction percentage increased as the carbon chain length of the PFAS increased. A higher number of interactions among the residues in Region 2 results in a more stabilized helix–loop–helix structure in the presence of longer PFAS only. The importance of Region 2 for the thyroid hormone synthesis was observed in a recent study where the crystal structure of bovine TG was obtained after the formation of T4 hormone.^17^ Upon comparison of hTG and bovine TG with T4, one significant difference was observed for Region 2: to allow for T4 formation, Region 2 was shifted, and three residues from the helix were unfolded and became part of the loop: S2534, S2535, and K2536. These are the residues that formed new hydrogen bonds in the presence of longer chain PFAS; in essence, the binding of longer chain PFAS triggers the formation of more interactions within Region 2, making it more rigid. Hence, by preventing the required flexibility, PFAS would be able to interfere with thyroid hormone synthesis.

The interaction pattern observed for Region 3 is similar to that for Region 1. While the interactions observed in the apo system were protected in most PFAS-bound simulations, the percentages were generally higher in the presence of PFAS (Table S13). In general, however, the hydrogen bond percentages did not show significant interaction differences between the apo simulations and PFAS simulations within this region.

One interesting observation for Region 3 is that the orientations of PFSA compounds were usually toward the residues within this region (Figure S7); however, PFCA compounds showed preferences toward the Lysine residues in Region 2. This orientation preference, as explained in the following section, results in a characteristic distribution of distance and angles between ITY residues in the presence of PFCA and PFSA (Figure S5).

### Impact of PFAS Binding on ITY Orientations

**Figure 2:**
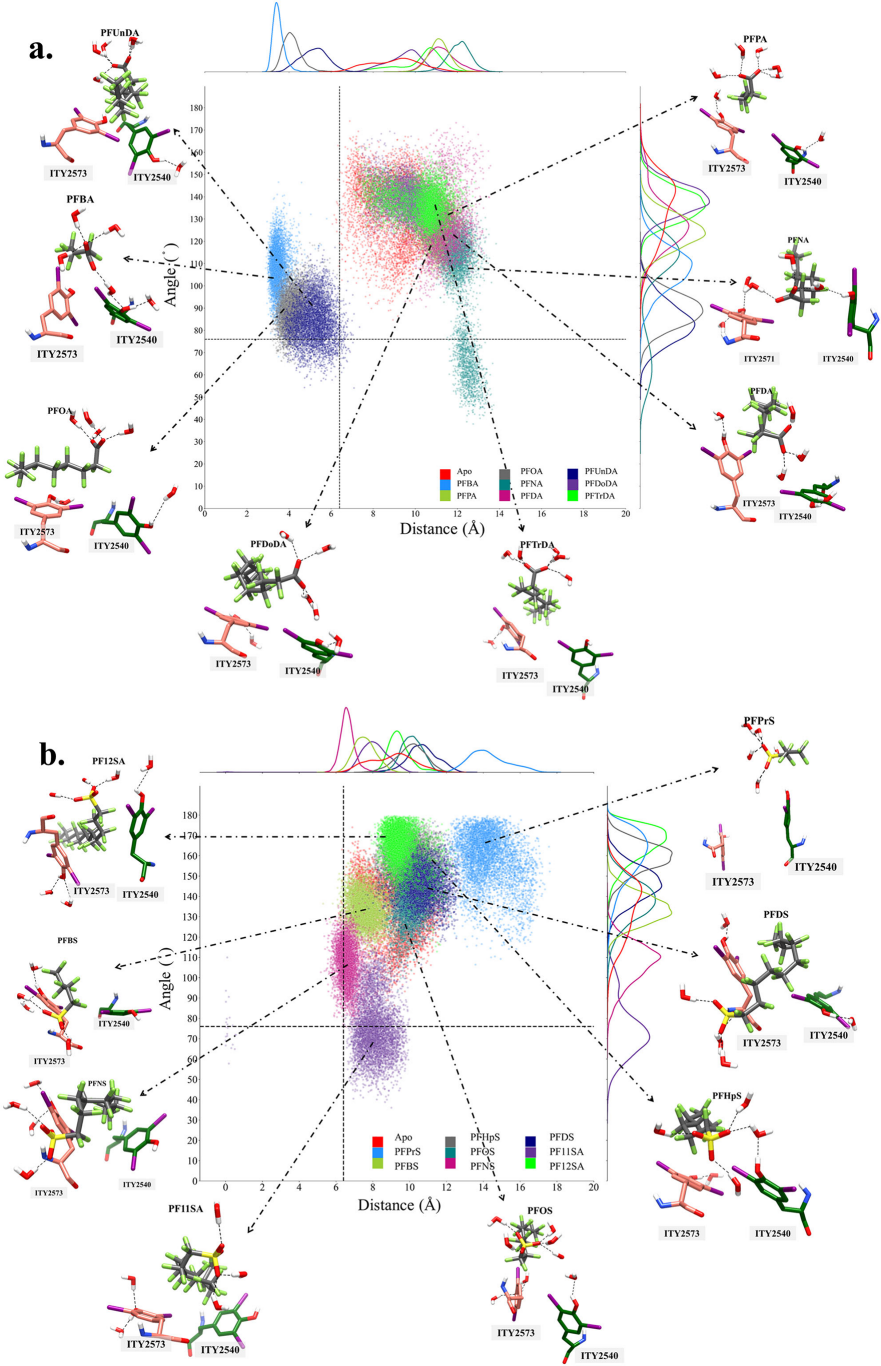

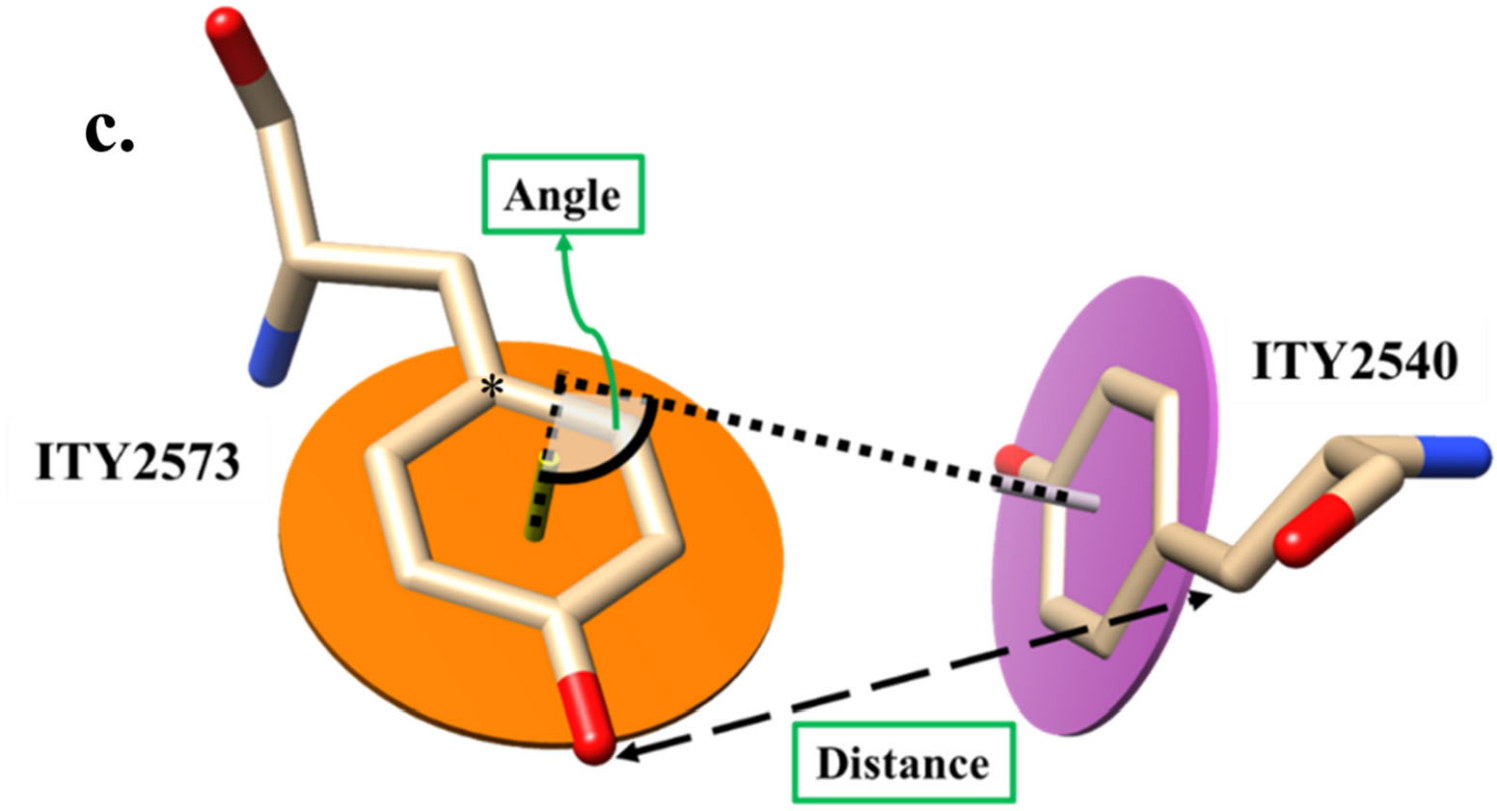
Angle/distance distribution of ITY side chains when PFCA (a) or PFSA (b) are present in the pocket. The angles are calculated between the normal vectors of the planes, as described in (c) and Figure S5. The most dominant orientations for each PFAS are also shown. The horizontal and vertical line intersection indicates the angle/distance calculated from the cryo-EM orientation (6.4 Å and 76°).

As the disturbance of thyroid hormone levels has been identified as one of the health consequences of PFAS exposure, understanding how the presence of PFAS could affect thyroid hormone synthesis in the investigated hormonogenic site is fundamental. The proposed mechanism for T4 synthesis indicates that the acceptor and donor ITY residues should be within ∼6 Å and nearly be parallel to one another, based on the available cryo-EM structures of hTG.^15^ While the angle between the ITY planes provides insight about the respective positioning of the side chains of these hormonogenic residues, the distances between the donor and acceptor atoms are also important features in assessing thyroid hormone formation. Therefore, the distance between the oxygen from the donor ITY2540 and the carbon from the acceptor ITY2573 were tracked for all simulations. The angle between the ITY side chains was also calculated, and their distributions as well as the dominant orientations of residues are shown in Figure 2.

The angle/distance distribution plots show that the PFCA and PFSA compounds impact the ITY orientation. The ideal positioning of ITY residues in Site B which would allow for the formation of T3 and T4 hormones has a ∼76° angle and ∼6 Å distance, based on the available cryo-EM structure of human thyroglobulin (Figure 2). The presence of PFAS generally limited the conformational space of ITY residues, in terms of the distance and angle tracked here. The apo system has a single peak at ∼145° along with a shoulder at ∼120° with a wide distribution. The distance range of the apo simulation was observed to be between ∼6–14 Å. PFCAs had a broader distance distribution (∼3–14 Å), while PFSA compounds displayed a narrower one, around 6 to 12 Å, with the exception of PFPrS. The correlation between the fluorinated carbon chain length and angle, however, shows different preferences between PFCA and PFSA compounds. The smallest angle in the distribution observed for PFCA was ∼60° (small peak of PFNA), and it was ∼70° for PFSA (PF11SA). On the other hand, the largest angles observed were for PFDoDA and PFTrDA (∼140°), and PFPrS, PFHpS, and PF12SA (∼170°) among the PFSA compounds. Overall, the smallest angle/distance distribution among PFCA was observed for PFBA, PFOA, and PFUnDA, while among PFSA, it was PFBS, PFNS, and PF11SA.

The two clusters formed by PFCA compounds (Figure 2) can be distinguished by a distance threshold of 6 Å. Only three PFCA compounds had distances smaller than 6 Å: PFUnDA, PFBA, and PFOA. However, only in PFBA bound simulations, which is a weak binder, do ITY residues show a distance/angle distribution that would allow for the formation of thyroid hormones. On the other hand, PFOA has an average binding strength, as per MM-PBSA energies, and it has strong interactions with the ITY2573 residue. Similarly, PFUnDA has strong binding energy and strong interactions with ITY2540. The strong interactions with ITY residues could prevent them from forming T3 and T4 thyroid hormones. The other cluster seen in Figure 2(a) has a large distance (8–14 Å) and angle (100–140°). PFNA, among those compounds, showed a strong peak around 120° with a smaller peak around 70°.

The interesting fact about PFNA interactions that played a role in the bimodal distribution is the stronger interactions with ITY2540, instead of ITY2573, as mentioned above (Table S6). The interaction preference also contributes to the “sandwiching” behavior that was seen in PFNA simulations, where PFAS places itself in-between two ITY residues (Figure S7). Furthermore, the stronger MM-PBSA binding energy of PFNA can also be attributed to the sandwiching interaction. Other PFCAs have mainly stronger interactions with ITY2573 through their tail groups, and do not show “sandwiching” behavior. Among the PFSA species, PFNS and PF12SA had a similar interaction type where the intercalation between ITY residues happened (Figure S7). In this case, however, PFNS exhibited strong interactions with ITY2573, while PF12SA had interactions with both ITY, with comparable strengths (Table S6).

Many of the PFAS bound systems did not show any distances and/or angles closer to those observed in the cryo-EM structure, except for PFUnDA, which had an ∼80° angle and ∼5 Å distance. For the PFSA, no system had values close to those of the experimental structure, indicating that the presence of various PFAS near hormonogenic site B can prevent the conformational space that would allow the formation of thyroid hormones. Furthermore, based on our analysis of the investigated PFAS-bound systems, the degree in which the PFAS can impact this conformational space depends on (i) the interaction mode of PFAS with the surrounding residues, including ITYs, (ii) the length of the tail group of PFAS, and (iii) the hydrogen bond interactions of headgroup of PFAS.

## Discussion

The binding energies indicate that PFSA molecules have stronger interactions with the investigated site than with PFCA compounds, as shown in Figure 1(c). Furthermore, there is a chain-length-dependent effect on the binding strength, although this dependence is not completely linear. As the chain length increased from three to eight or nine fluorinated carbons (PFNA and PFNS, respectively), the binding energies showed a linear increase. And as the chain gets longer than eight or nine carbons, however, there is a drop in the binding strength, indicating that PFAS with eight and nine carbons can impact the hTG Site B by binding more strongly than shorter chain PFAS and forming key interactions with surrounding residues.^7,12,18,19,31^ A 2023 study by Vollmar et al. suggests that PFOS and PFOA have the potential to disrupt the T4 levels.^33^ Our study for the first time shows that the disruption by PFAS occurs through binding to the hTG protein and, thus, interferes with the thyroid hormone synthesis.

The presence of PFAS, overall, causes the conformational space of the distance and angle between the two ITY residues to narrow as compared to the distribution observed for the apo system. While ITY residues do require the thyroid peroxidase (TPO) enzyme to form the thyroid hormones through a mechanism that is still unknown, the proximity and relative orientation of ITY residues are still important for successfully producing T3/T4 hormones.^15,16,34^ The distance and angle between the two ITY residues in the majority of PFAS-bound systems (Figure 2(a,b)) did not match the distribution observed in the cryo-EM structure. Moreover, the influence of the PFCA compounds on the conformational space of ITY residues suggests a wider range of distances compared to that of PFSA molecules, indicating that these two series formed interactions with different residues. While PFCA head groups prefer to orient toward ITY2540, PFSA compounds pointed toward the loop structure near the binding site. The interactions of PFAS also significantly impacted the distance and angle distributions significantly. PFNA and PFNS, for instance, showed a particular “sandwiching” behavior between two ITY residues, that was not observed for any other PFAS investigated. These two PFAS also had strong hydrogen bonds with the surrounding residues. Together, these different types of interactions lead them to have strong binding energies and, consequently, have more pronounced adverse effects on thyroid hormone synthesis on Site B.

The local interaction changes within the binding area indicate that the longer chain PFAS could lead to a more rigid helix structure in Region 2. A comparison with a recent cryo-EM structure of the bovine TG with T4 formed in Site B shows that there is a shift in Region 2 associated with the formation of the thyroid hormone.^17^ Therefore, for the first time, we suggest that the changes to the hydrogen bond network within Region 2 upon long-chain PFAS binding could inhibit the required motion for the formation of thyroid hormones.

## Conclusion

As the linkages between PFAS exposure and health problems are increasing and the governments such as in the United States and the European Union are proposing restrictions on PFAS production due to these adverse health effects, a detailed molecular understanding of PFAS toxicity through computational methods is necessary to establish effective mitigation strategies. To the best of our knowledge, this work is the first of its kind to investigate the influence of PFAS binding to Site B of hTG and the potential impact of PFAS on thyroid hormone synthesis by causing rigidity in the binding region. We observed that PFAS with eight to nine carbons with a distinct binding mode showed higher binding energies. The longer chain PFAS, on the other hand, resulted in a change in the rigidity of Region 2, which is important for thyroid hormone synthesis. Understanding these governing factors of PFAS toxicity on thyroid hormone synthesis can help enable the development of effective mitigation strategies and understand harmful influences of PFAS in humans better.

## Methodology

### System Preparation

The dimeric human hTG protein atomistic structure (PDB ID: 6SCJ, 3.6 Å resolution) was obtained from the RSCB Protein Data Bank.^15^ The missing loops of the structure were modeled using the I-TASSER server separately by including ten amino acids from each end of the missing loops.^35^ The prepared structure was solvated and then minimized using Amber20 as described in the Simulation Details section (RMSD is shown in Figure S8).

### Docking Procedure

The initial step of this investigation involved selecting a list of carboxylic PFAS (PFCA) and sulfonic PFAS (PFSA) with carbon chains varying from four to 12; their structures are provided in Table S1. Molecular Operating Environment 2022 (MOE 2022.02) software was used for the docking procedures and protonation state determination.^36^ The minimized hTG dimer structure was used for the docking procedures. The binding pocket was defined by using a pharmacophore docking strategy to place the PFAS head groups near hormonogenic Tyr residues. The pharmacophore consisted of two features to place the headgroup. For the short-chain PFAS, a docking procedure with no pharmacophore was also performed. The pharmacophore was used for the initial placement with the London dG scoring function to obtain 100 poses, which were further refined to five poses with an induced fit method and Generalized Born Volume integral/Weighted Surface Area (GBVI/WSA) scoring function.^36^ The highest scoring poses for each PFAS were selected for Molecular Dynamics (MD) simulations. For the docking procedure without pharmacophore placement, the Triangle Matcher method was used for the initial PFAS placement. Both monomeric and dimeric structures were considered in the modeling of the binding of PFAS to Site B in the hTG protein to understand the effects of the dimer structure. The binding energies of the PFPA and PFBA compounds to the dimer hTG structure are reported in Table S14.

### Simulation Details

The dimer hTG apo, monomeric apo, and PFAS-bound hTG monomeric systems were prepared using the tleap module of Amber20/AmberTools22 software.^37^ The partial charges of PFAS compounds and iodinated Tyr residues (ITY) were calculated using the AM1-BCC method as implemented in the antechamber module of AmberTools22 with gaff2.^38,39^ The protein, PFAS, and waters were modeled using ff14SB, gaff2, and TIP4P-EW force fields, respectively.^37−41^ NaCl ions were added to obtain 0.15 M salt concentration to mimic the natural environment. On average, a monomeric system consisted of ∼680,000 atoms, while a dimeric system had ∼1,020,000 atoms, including solvent molecules.

The minimization and heating steps were performed in a stepwise fashion as follows:

(i) The minimization was done in four steps with the following restraints (100, 50, 10.0 kcal mol^–1^ Å^–2^), with each step having 20,000 cycles.
(ii) The systems were heated up from 0 to 20 K in 160 ps with a 3 kcal mol^–1^ Å^–2^ restraint applied on all atoms. Then, the systems were heated to 200 K for 250 ps with restraints applied to the backbone atoms only, followed by a short equilibrium simulation at 200 K for 200 ps with no restraints. Finally, heating to 300 K was done for 900 ps with no restraints applied.
(iii) Before the production step, a 500 ps long equilibrium simulation was performed at 300 K.

The minimized and equilibrated structures were simulated for 20 ns at 300 K and 1 atm using 1 fs timesteps with the SHAKE algorithm.^42^ A duplicate set of simulations was performed by reinitializing the velocities after the heating step. The Langevin thermostat and isotropic position scaling were selected for the temperature and pressure controls, respectively.^43,44^ All simulations were performed using the *pmemd.cuda* module as implemented in the Amber20 suite.^37,45^

### Analysis Details

The binding energies were calculated by selecting every tenth frame for the last 5 ns of simulations, resulting in a total of 500 frames for a single simulation. The Molecular Mechanics–Poisson–Boltzmann Surface Area/Generalized Born Surface Area (MM-PBSA/GBSA) method was used to estimate the binding strengths of the PFAS, as implemented in Amber20/AmberTools22.^46^ For MM-GBSA calculations, GB^OBC^ was used with α, β, and γ parameters with values of 1.0, 0.8, and 4.8, respectively. The radii2 effective Born radius was selected as implemented in Amber20.^37,47^ The dielectric interface was implemented with the level set function.^37^ The salt concentrations were selected as 0.1 M for both MM-GBSA and MM-PBSA calculations. As the focus of this work is not the exact estimation of the binding energies, but rather to provide a ranking of the binding strengths, MM-PBSA/GBSA methodology is useful in providing insight about binding pockets with partial solvent exposure.^48−54^ A longer simulation was performed for the PFNA/hTG system in duplicate to assess the convergence of the MM-PBSA energies, and the results are shown in Figure S9. Our observations from the extended PFNA simulations support the initial proposed values, since the new values fluctuate within approximately ±2.5 kcal/mol, corresponding to the standard deviation.

The root-mean-square deviations (RMSDs), per-residue root-mean-square fluctuations (RMSFs), and hydrogen bond analysis were calculated using the *cpptraj* module from AmberTools22 with the default parameters.^55^ The per-residue decomposition energies were calculated by taking the nonbonded interactions into account for the residues within 10 Å of the PFASs. Clustering was performed to obtain the most dominant orientations of the ITY residues and PFAS using a hierarchical agglomerative algorithm with an epsilon value of 3.0. The total energy convergence as well as the structural convergence of the simulated systems were considered, and the last 5 ns of the trajectories were utilized for all analysis.

Clustering of the trajectories was performed using the dbscan (Density-Based Spatial Clustering of Applications with Noise) method as implemented in the cpptraj module, using six minimum samples and an epsilon value of 2.^55^ The number of minimum samples was determined by a k-distribution plot. Only the last 5 ns of the trajectories were considered for this analysis.

### Orientation of Hormonogenic Tyr Residues

The positions of ITY residues were clustered, and the angle and distance between them were measured for the last 5 ns of each simulation, as shown in Figure S5. The distance between the center-of-mass of the side chain atoms of ITY residues and the distance between the reactive atoms were measured. To measure the angle between the ITY residues, a plane for each ITY residue was described by two vectors: each extending from the CG atom to the iodine atoms (Figure S5). Then, the angle between the two planes was calculated to estimate the relative orientations of the ITY residues.

## Data Availability

The authors declare that data supporting the findings of this study are available within the paper and its Supporting Information. The starting structures are uploaded in Zenodo (DOI: 10.5281/zenodo.10277855).

## Supporting Information Available

The structures of PFAS used in this study. RMSD time-series plots, per-residue RMSF plots, hydrogen bond percentages, and per-residue interaction energies time-series of reported simulations. Distance-angle distributions of ITY residues for reported simulations. Dominant orientations and interactions of each PFAS in the reported simulations (PDF)
