# Supplementary material for: Per- and Polyfluoroalkyl (PFAS) Disruption of Thyroid Hormone Synthesis
Source: ACS Omega. 2024 Sep 10;9(38):39554–63. doi: 10.1021/acsomega.4c03578 (PMC11425649; doi:10.1021/acsomega.4c03578)
Supplement: Supplementary file 1 — ao4c03578_si_001.pdf [file ao4c03578_si_001.pdf]

Supporting Information

for

Per- and Polyfluoroalkyl (PFAS) Disruption of Thyroid Hormone Synthesis

Semiha Kevser Bali[a], Rebecca Martin[a], Nuno M. S. Almeida[a], Catherine Saunders[a], Angela K. Wilson[a]*

[a]Michigan State University, Department of Chemistry, East Lansing, MI 48864, U.S.A
*akwilson@msu.edu

Table of Contents

Tables:

Table S1. The list of PFAS used in this work, the number of fluorinated carbons, and their 2D structures. 3
Table S2. The RMSD time-series of apo system and carboxylic acid PFAS simulations........4
Table S3. The RMSD time-series of sulphonic acid PFAS simulations. 7
Table S4. The total energy of carboxylic acid PFAS simulations........9
Table S5. The total energy of sulphonic acid PFAS simulations........11
Table S6 . Average per-residue decomposition energies for each PFAS simulation of non-polar pocket residues along with ITY residues. 13
Table S7. Average per-residue decomposition energies for each PFAS simulation of polar pocket residues........14
Table S8. Average per-residue decomposition energies for each PFAS simulation of basic pocket residues. 15
Table S9. Average per-residue decomposition energies for each PFAS simulation of acidic pocket residues........15
Table S10. Hydrogen bond percentages of PFAS head group oxygen atoms. Res. ID: Residue ID of the amino acids........16
Table S11. Average hydrogen bond % fractions of Region 1. From left to right, the fluorinated carbon chain length increases........17
Table S12. Average hydrogen bond %100 fractions of Region 2. From left to right, the fluorinated carbon chain length increases........18
Table S13. Average hydrogen bond 100% fractions of Region 3. From left to right, the fluorinated carbon chain length increases........19
Table S14. The MM-GBSA/PBSA binding energies of PFBA and PFPA with dimer hTG protein. The compounds did not form strong interactions with the binding site residues and did not reside within the region........20

Figures:

Figure S1. Formation of T4 by hormonogenic Tyrosine residues. Iodine is shown with yellow spheres..21
Figure S2. Per-residue RMSF plot of first simulation set. 21
Figure S3. Per-residue RMSF plot of second simulation set. 21
Figure S4. The regions for which the hydrogen bond patterns were investigated. Region 1 is shown in blue and includes S675, Q676, P677, A678, G679, and S680 residues. Region 2 is shown in green and includes V2523, K2524, Q2525, F2526, E2527, E2528, S2529, R2530, G2531, R2532, T2533, S2534, S2535, K2536, T2537, A2538, F2539, and ITY2540. Region 3 is depicted in pink loop representation and has the following residues: H2568, S2569, T2570, D2571, D2572, ITY2573, A2574, S2575, F2576, S2577, and R2578. The rest of the Thyroglobulin protein is shown as cartoon in grey color. PFOA is shown in wire representation, and ITY residues are shown in stick representation..................................22
Figure S5. Representation of angle and distance measurements between ITY2540 and ITY2573. The residues are shown in stick representation, and the planes, shown as disks, are created by considering the side chain ring atoms. The normal of planes are shown as sticks. The distance between the OH atom of ITY2540 and CB atom of ITY2573 is shown with a dashed black arrow. CG atom is indicated by asterisk (*) on ITY2573, for reference. The structure of ITY residues is taken from the cryo-EM structure (PDB ID: 6SCJ). The distance calculated for the structure is 6.4 Å and the angle is 76 °..................................22
Figure S6. The kernel density plot of the distribution of calculated angles between the ITY residues. Above: PFCA compounds, below: PFSA compounds. Apo system is shown in solid red line in both plots. 23
Figure S7. The dominant orientations of ITY residues and PFAS compounds, extracted by clustering the last 5ns of the simulations. The key residues that have either the highest/lowest interaction with PFAS or that make hydrogen bonds with PFAS were shown in stick representation. The coloring of the secondary structures was based on the scheme shown in **Figure S4.** PFCA: per-fluoroalkyl carboxylic acid, PFSA: per-fluoroalkyl sulphonic acid. The figure is obtained using Chimera...26
Figure S8. RMSD plot of apo hTG dimer protein is shown. The purple line represents the whole-protein backbone RMSD, and the green line represents the RMSD of the heavy atoms in the pocket residue. 26
Figure S9. The MM-PBSA binding energies for PFNA compound. The simulations were performed for 60 ns in duplicate and the binding energies were calculated for every ns, then averaged for 5 ns windows. 16-20 ns range is the value reported in the **Figure 1(c)**..27
Figure S10. Surface Area calculations of ITY residues in the presence of PFAS. 28

Table S1. The list of PFAS used in this work, the number of fluorinated carbons, and their 2D structures.

Carboxylic PFAS Name	# of Fluorinated Carbons	Structure	Sulphonic PFAS Name	# of Fluorinated Carbons	Structure
**PFBA**	3	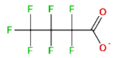	**PFPrS**	3	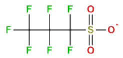
**PFPA**	4	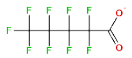	**PFBS**	4	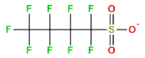
**PFOA**	7	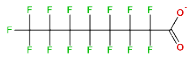	**PFHpS**	7	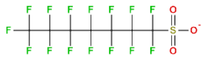
**PFNA**	8	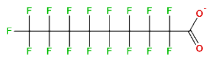	**PFOS**	8	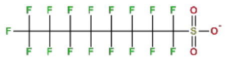
**PFDA**	9	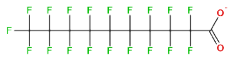	**PFNS**	9	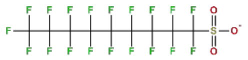
**PFUnDA**	10	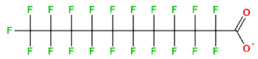	**PFDS**	10	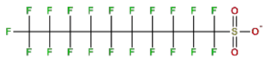
**PFDoDA**	11	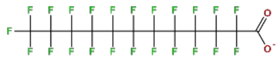	**PF11SA/PFUnS**	11	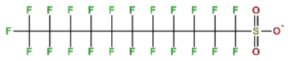
**PFTrDA**	12	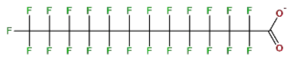	**PF12SA/PFDoS**	12	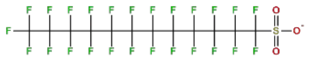

Table S2. The RMSD time-series of apo system and carboxylic acid PFAS simulations. The structural convergence analysis with RMSD (**Table S2-S3)** further indicated that although the hTG monomer had a high overall RMSD value, the local regions near the PFAS binding area had an average RMSD of ~2 Å RMSD and reached convergence as early as 8-10 ns of the simulations. RMSDs of the ITY residues and PFAS were also calculated for the 20 ns-long simulations. In most simulations, the RMSD values were 1-2 Å for ITY residues. PFAS showed a more diverse RMSD distribution overall; the short-chain PFAS, such as PFBA and PFPrS, had RMSD changes mainly characterized by moving away from the binding site. Furthermore, longer-chain PFAS, namely PF12SA, had a conformation change early in the simulation. The conformation differences observed for sulphonic PFAS were less than for carboxylic PFAS. Overall, the last 5 ns of the simulations were considered for further analysis, as shown by RMSD and total energy plots, and PFAS showed no significant conformation change during this simulation period.

**Simulation #1**	**Simulation #2**
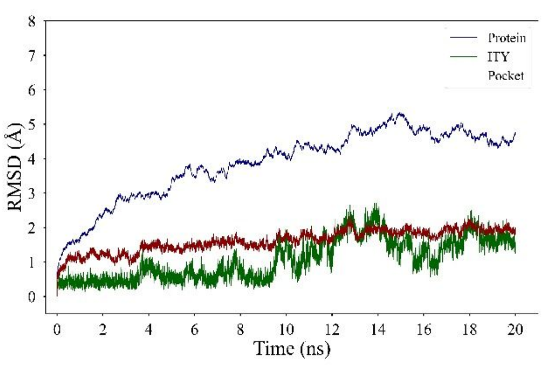	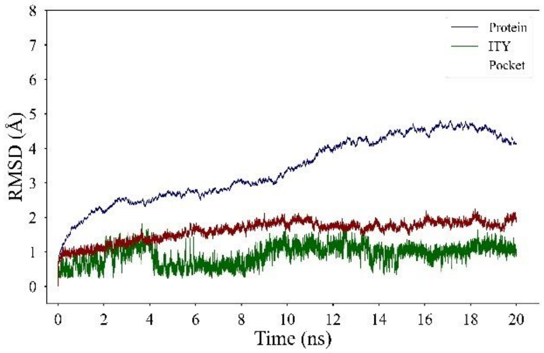
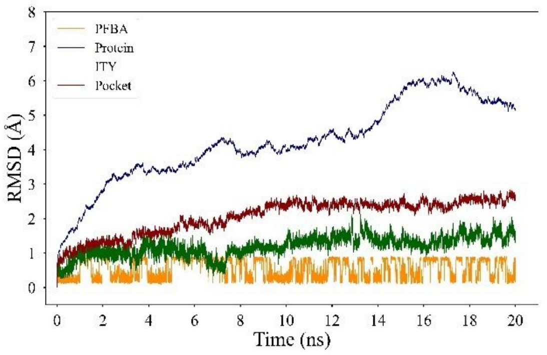	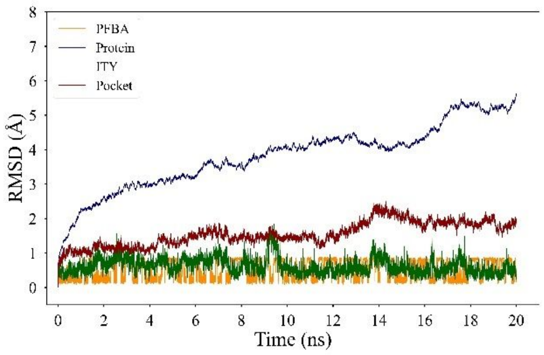
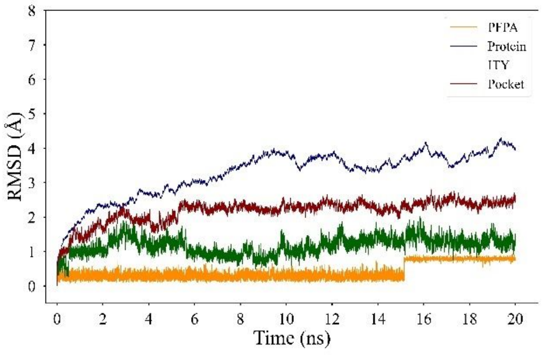	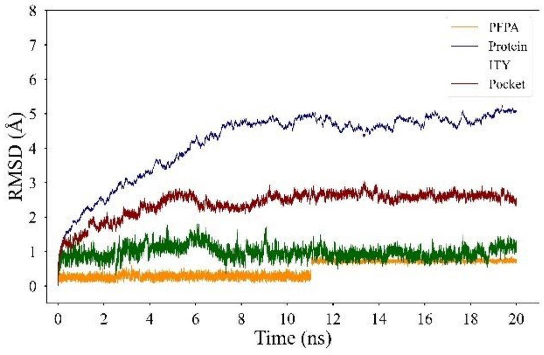

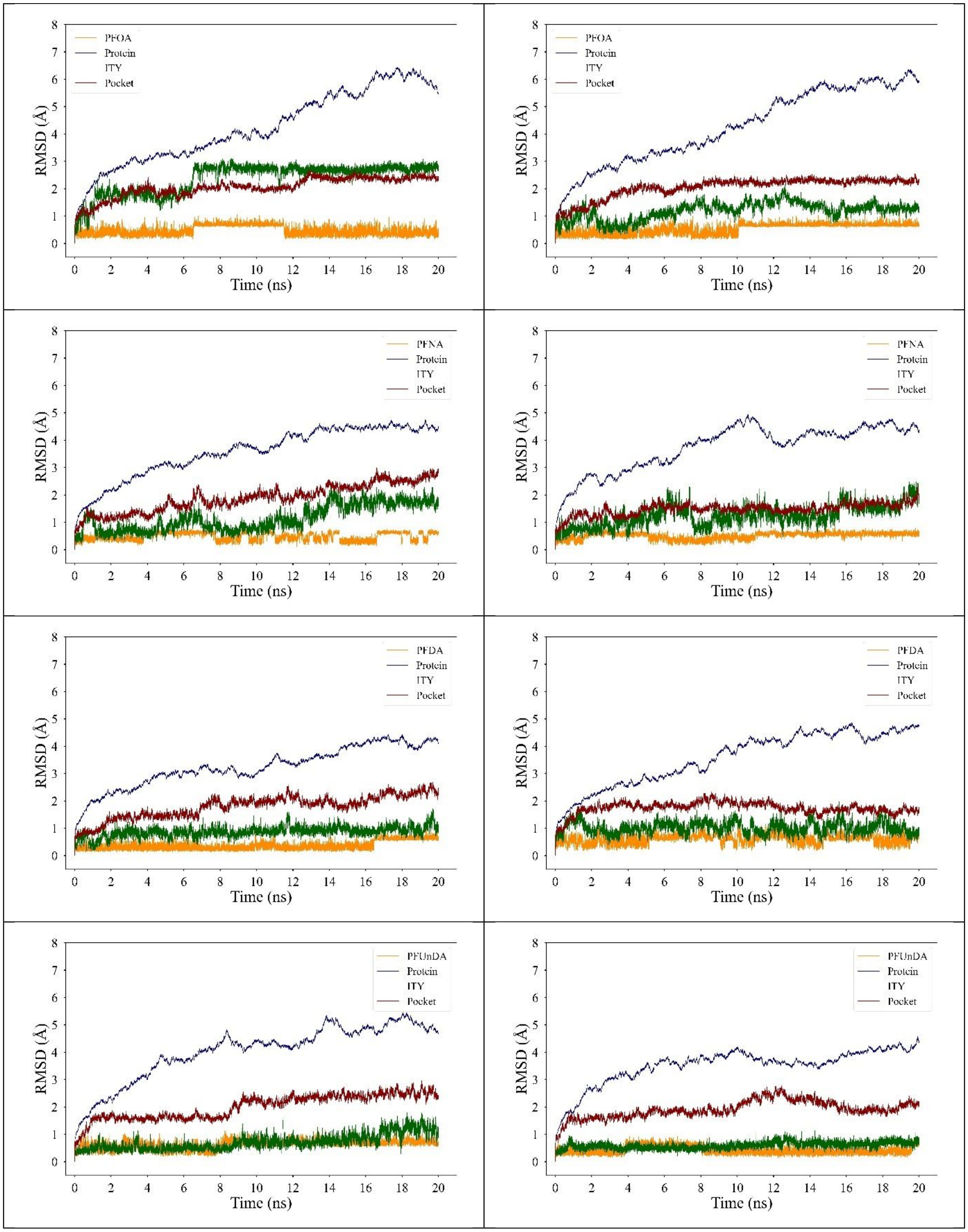
PFOA
Protein
ITY
Pocket
RMSD (Å)
Time (ns)
PFOA
Protein
ITY
Pocket
RMSD (Å)
Time (ns)
PFNA
Protein
ITY
Pocket
RMSD (Å)
Time (ns)
PFNA
Protein
ITY
Pocket
RMSD (Å)
Time (ns)
PFDA
Protein
ITY
Pocket
RMSD (Å)
Time (ns)
PFDA
Protein
ITY
Pocket
RMSD (Å)
Time (ns)
PFUnDA
Protein
ITY
Pocket
RMSD (Å)
Time (ns)
PFUnDA
Protein
ITY
Pocket
RMSD (Å)
Time (ns)

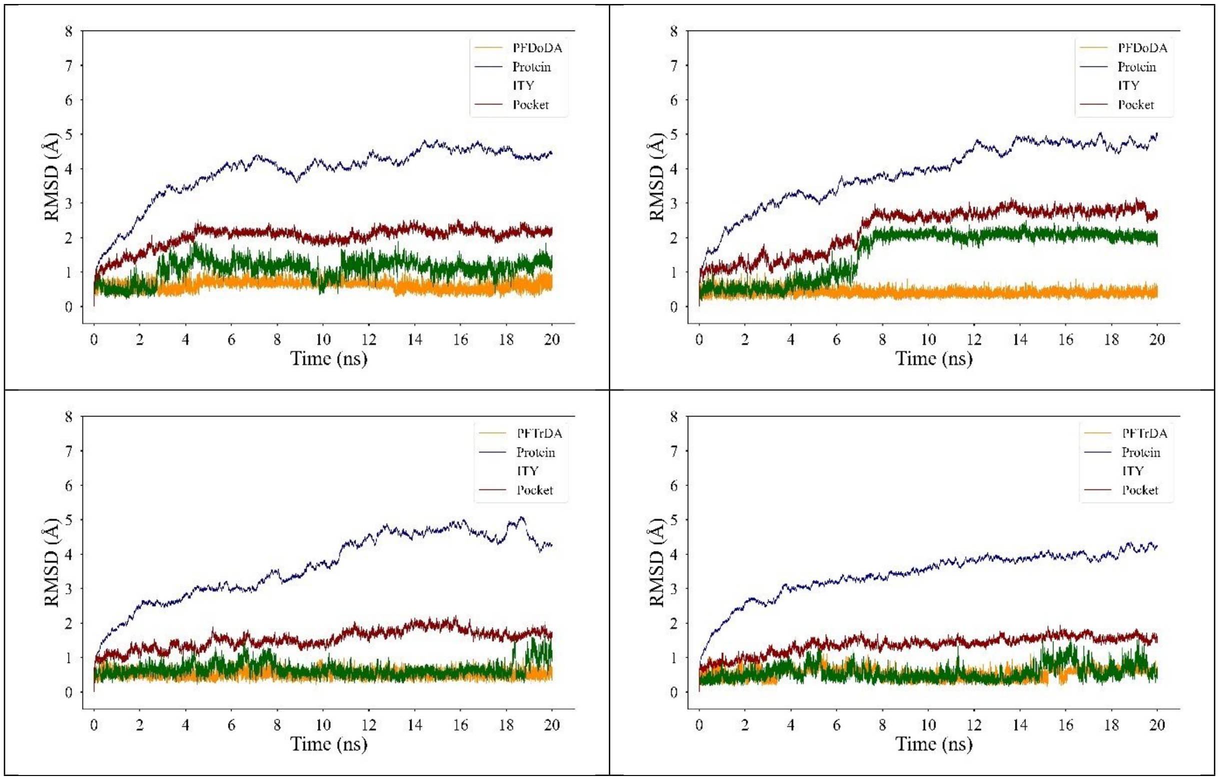
PFDoDA
Protein
ITY
Pocket
PFDoDA
Protein
ITY
Pocket
PFTrDA
Protein
ITY
Pocket
PFTrDA
Protein
ITY
Pocket
RMSD (Å)
Time (ns)

Table S3. The RMSD time-series of sulphonic acid PFAS simulations.

<table>
<tr><th>Simulation #1</th><th>Simulation #2</th></tr>
<tr><td>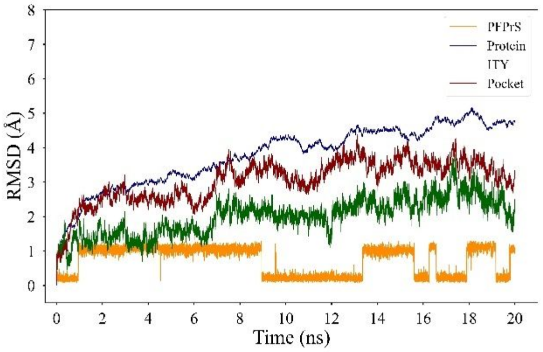
</td><td>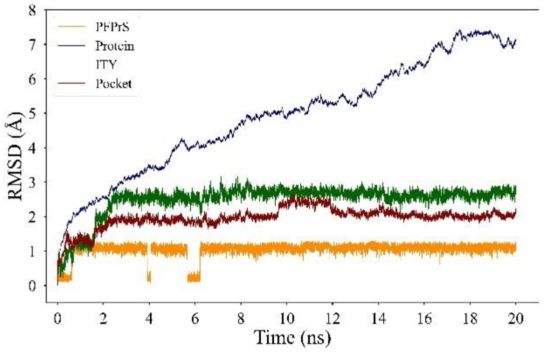
</td></tr>
<tr><td>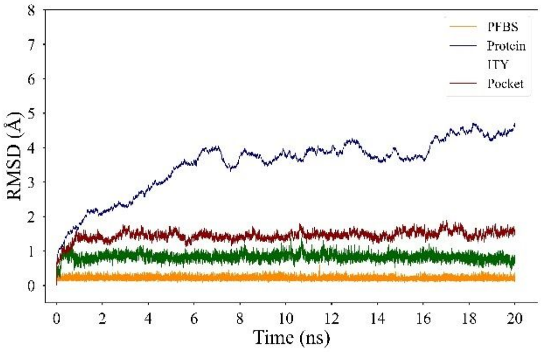
</td><td>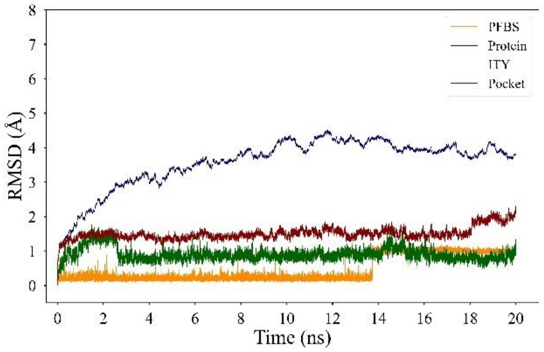
</td></tr>
<tr><td>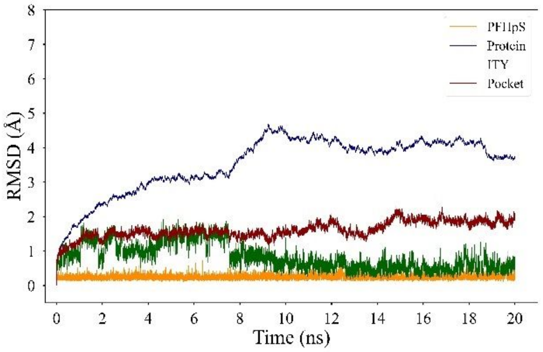
</td><td>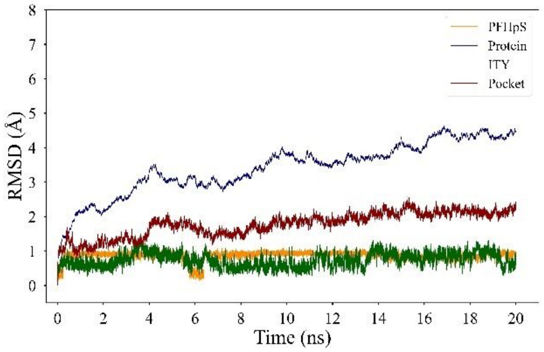
</td></tr>
<tr><td>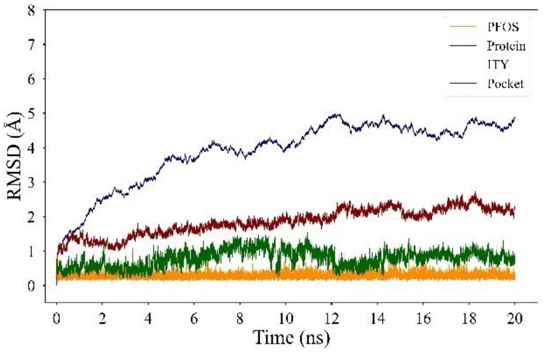
</td><td>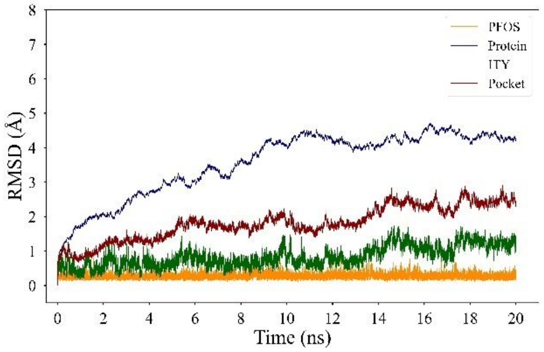
</td></tr>
</table>

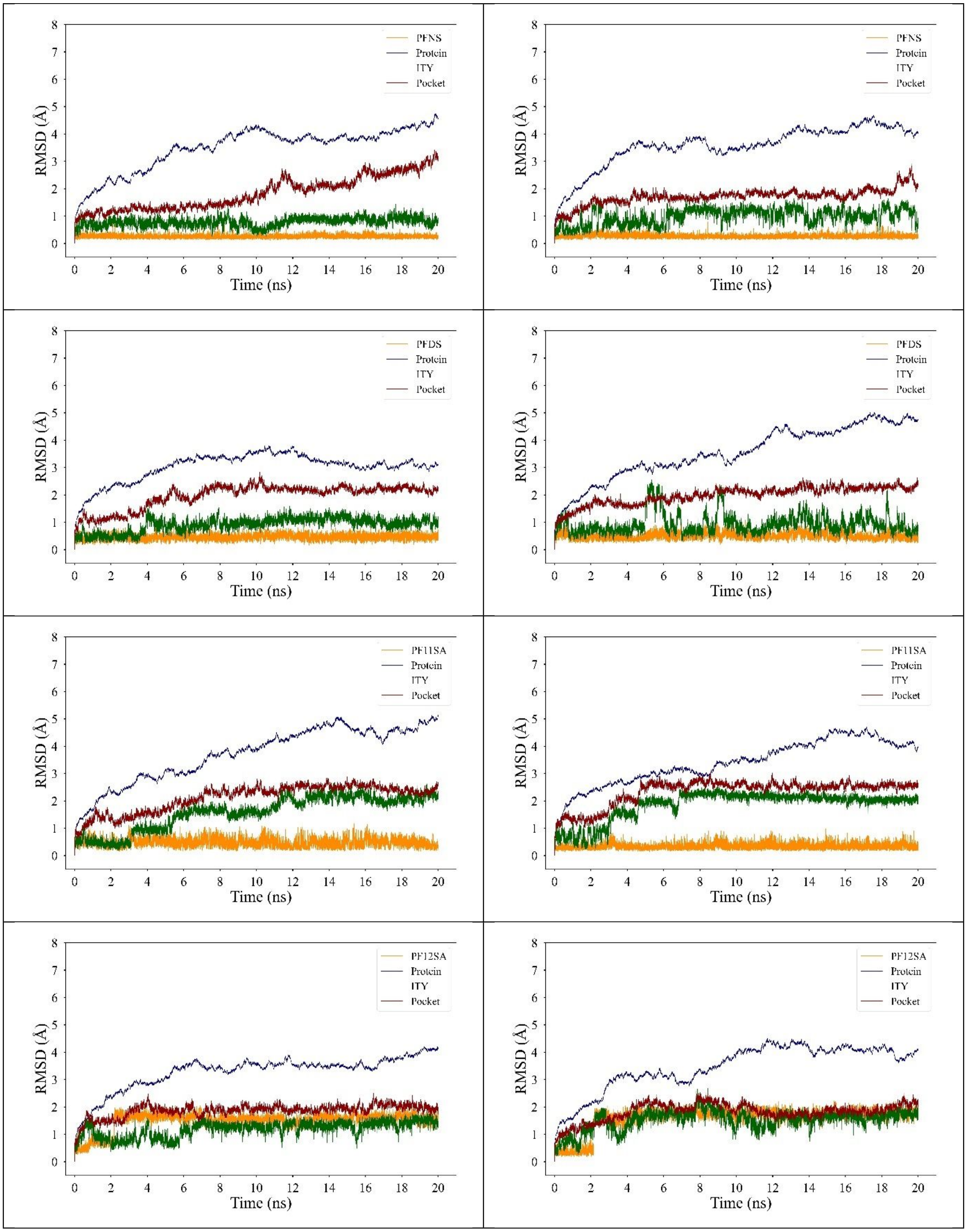
PFNS
Protein
ITY
Pocket
RMSD (Å)
Time (ns)
PFNS
Protein
ITY
Pocket
RMSD (Å)
Time (ns)
PFDS
Protein
ITY
Pocket
RMSD (Å)
Time (ns)
PFDS
Protein
ITY
Pocket
RMSD (Å)
Time (ns)
PF11SA
Protein
ITY
Pocket
RMSD (Å)
Time (ns)
PF11SA
Protein
ITY
Pocket
RMSD (Å)
Time (ns)
PF12SA
Protein
ITY
Pocket
RMSD (Å)
Time (ns)
PF12SA
Protein
ITY
Pocket
RMSD (Å)
Time (ns)

Table S4. The total energy of carboxylic acid PFAS simulations.

Simulation #1	Simulation #2
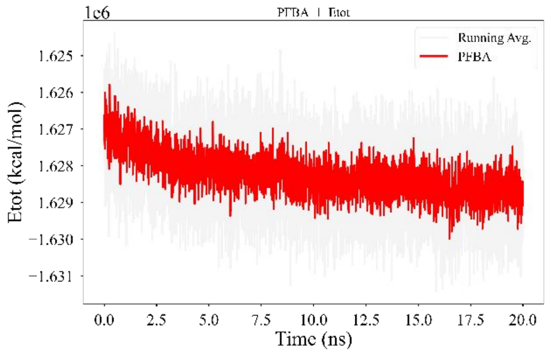	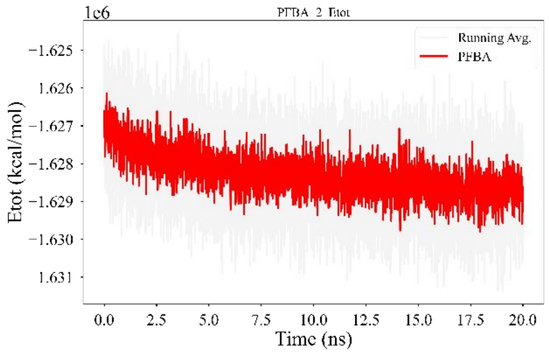
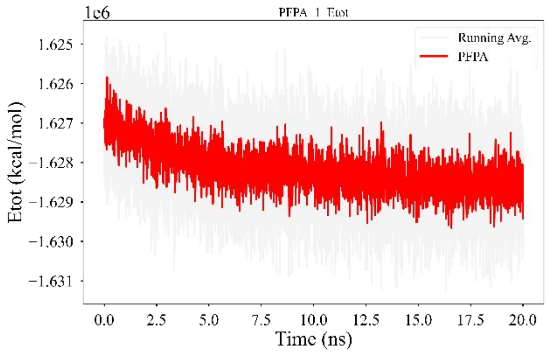	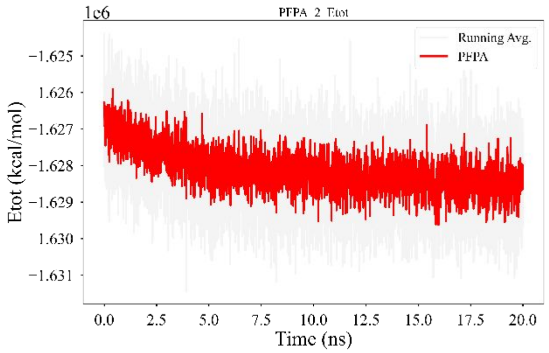
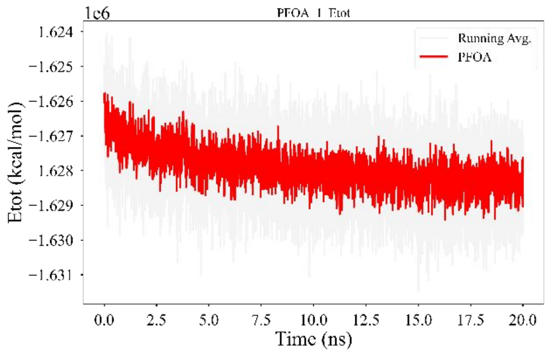	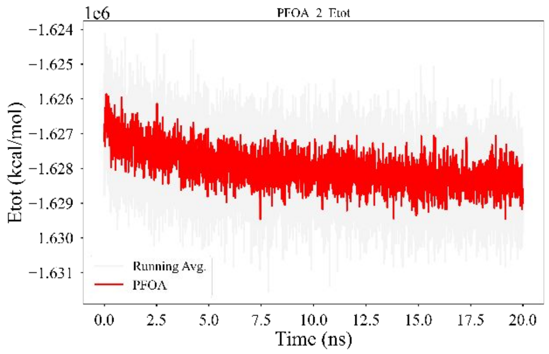
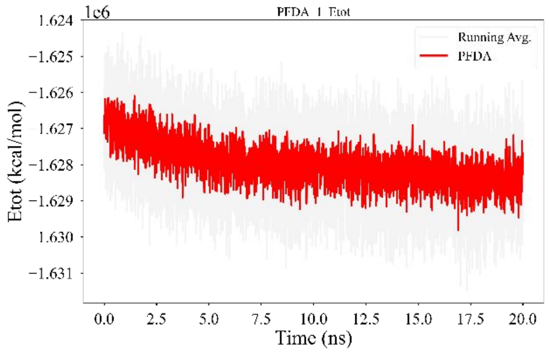	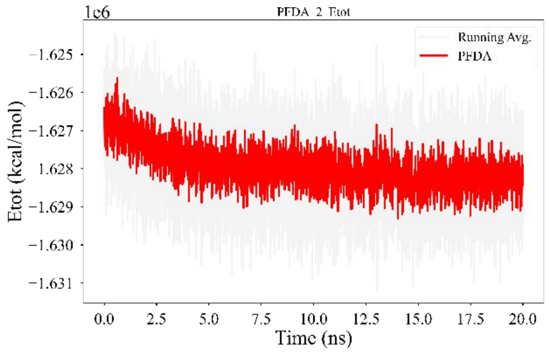

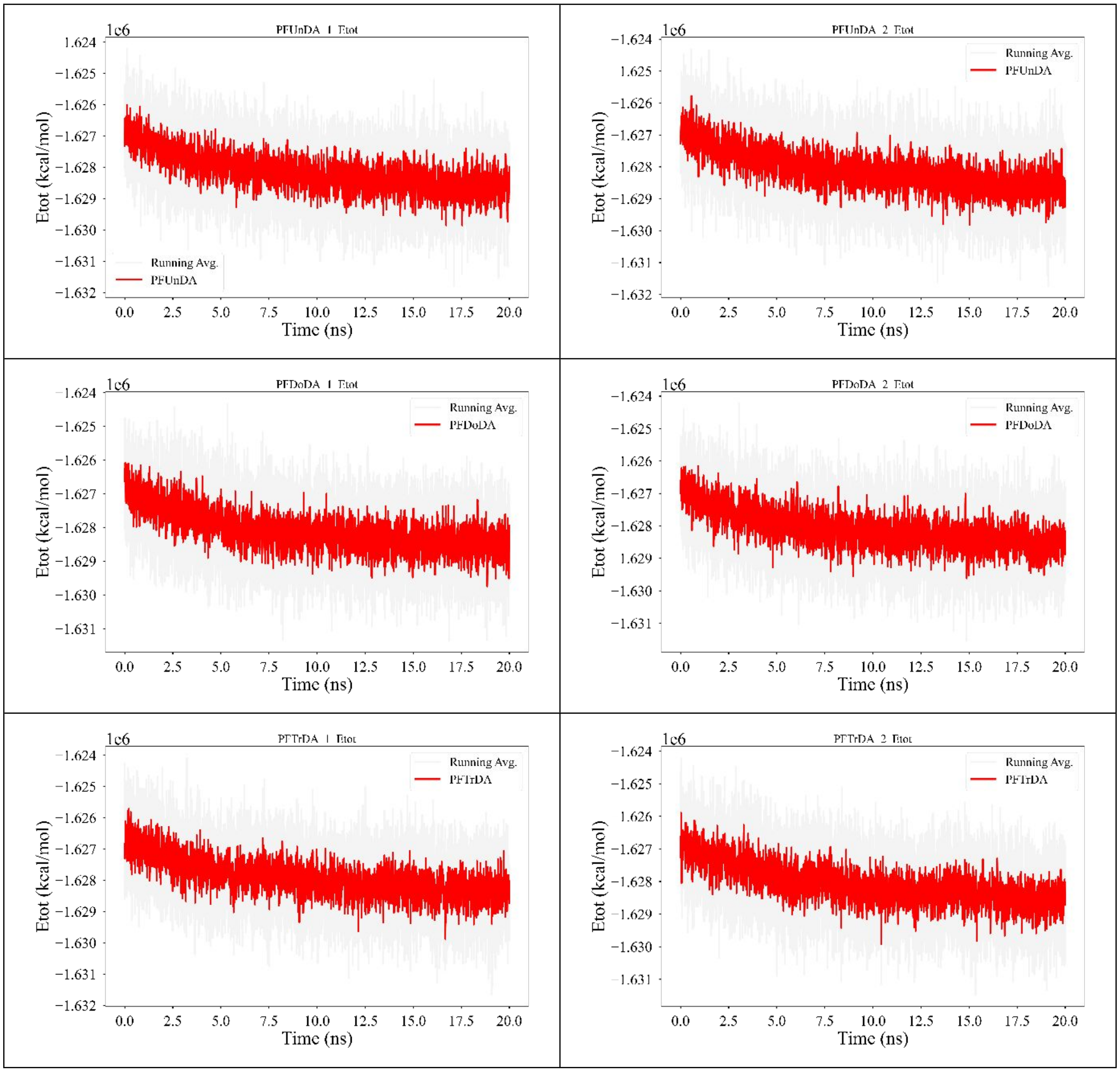
PFUnDA 1 Etot
Etot (kcal/mol)
Time (ns)
Running Avg.
PFUnDA
PFUnDA 2 Etot
Etot (kcal/mol)
Time (ns)
Running Avg.
PFUnDA
PFDoDA 1 Etot
Etot (kcal/mol)
Time (ns)
Running Avg.
PFDoDA
PFDoDA 2 Etot
Etot (kcal/mol)
Time (ns)
Running Avg.
PFDoDA
PFTrDA 1 Etot
Etot (kcal/mol)
Time (ns)
Running Avg.
PFTrDA
PFTrDA 2 Etot
Etot (kcal/mol)
Time (ns)
Running Avg.
PFTrDA

Table S5. The total energy of sulphonic acid PFAS simulations.

<table>
<tr><th>Simulation #1</th><th>Simulation #2</th></tr>
<tr><td>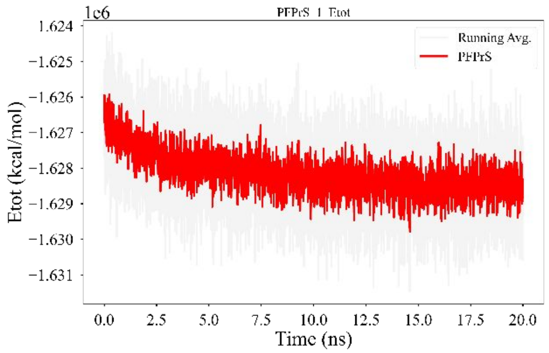
</td><td>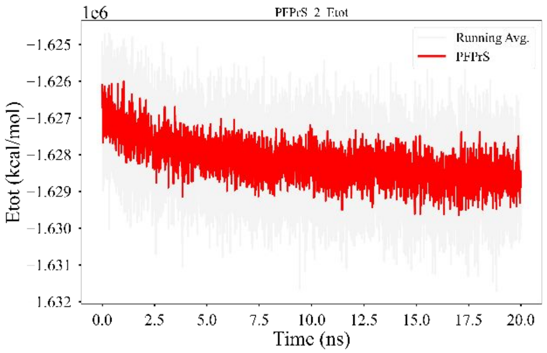
</td></tr>
<tr><td>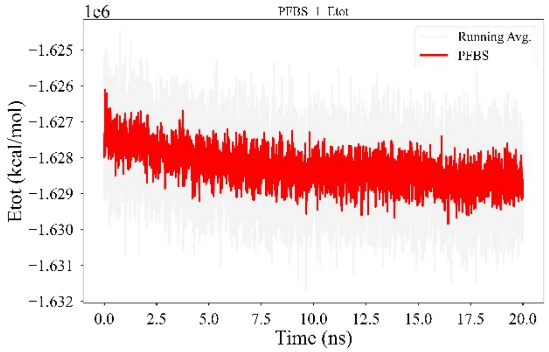
</td><td>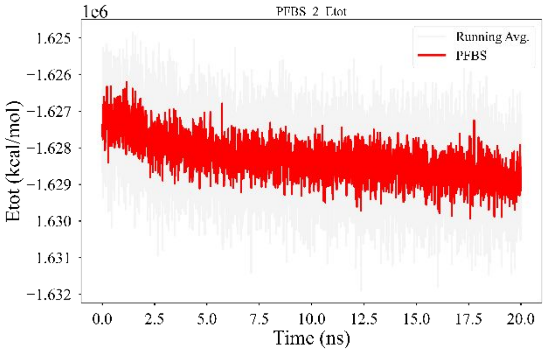
</td></tr>
<tr><td>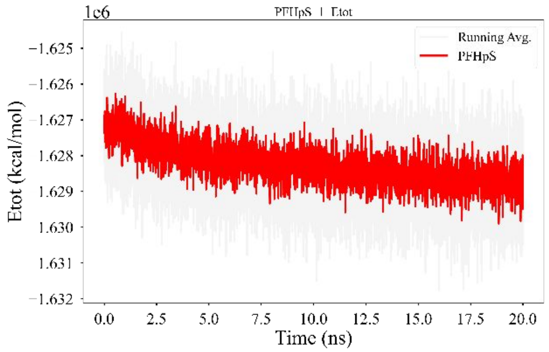
</td><td>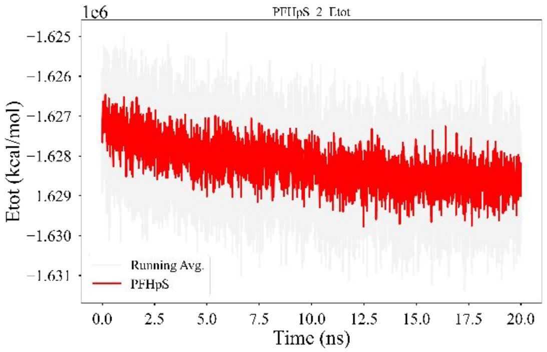
</td></tr>
<tr><td>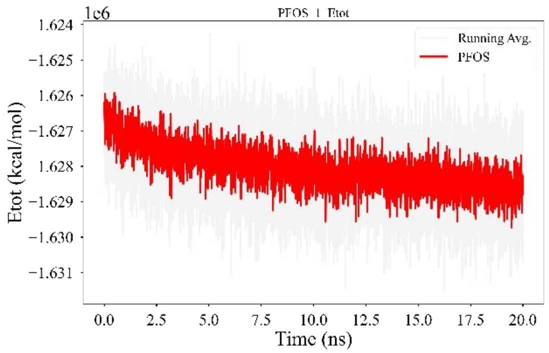
</td><td>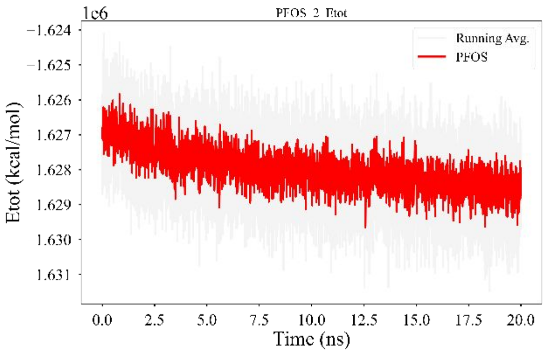
</td></tr>
</table>

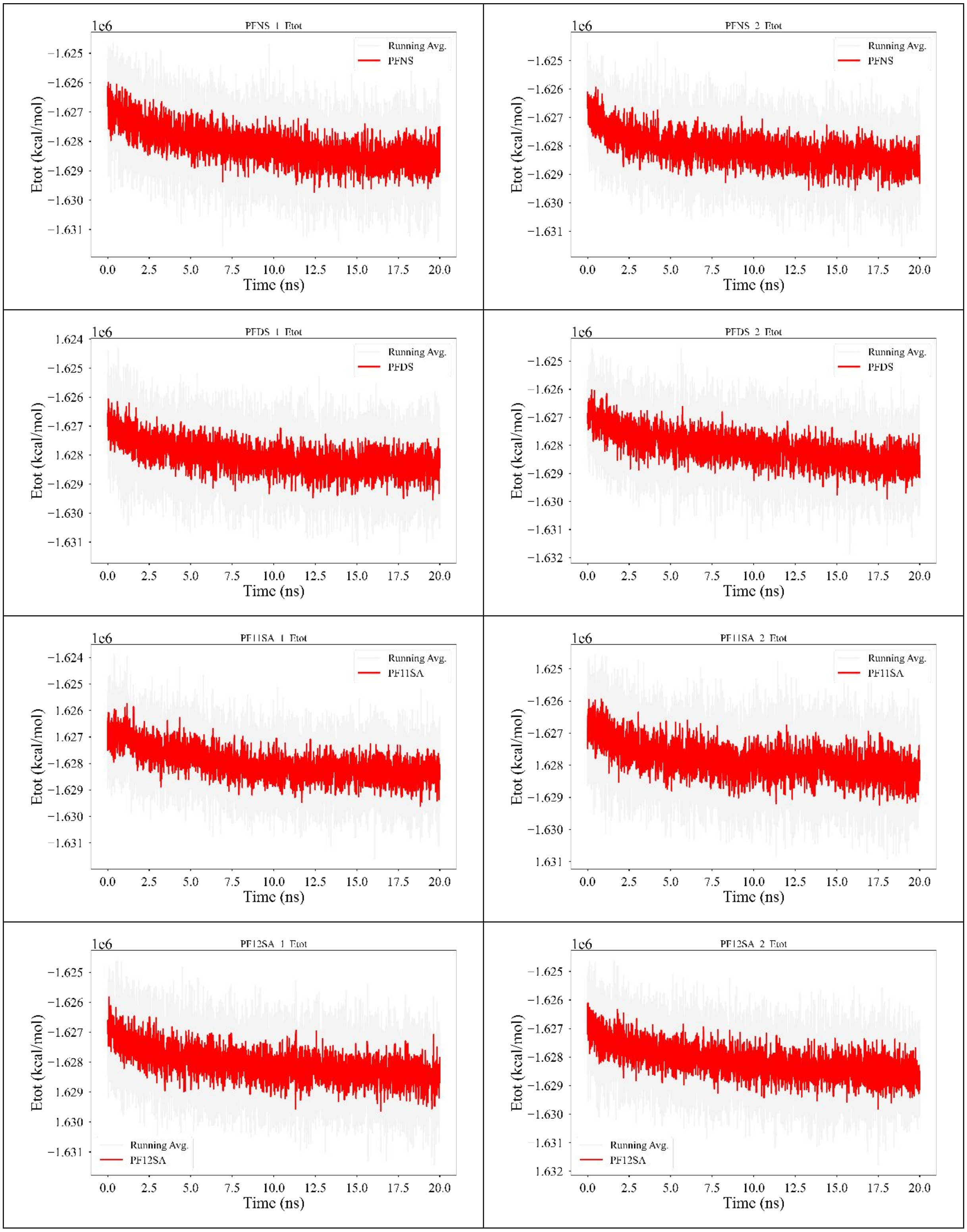
PFNS 1 Etot
PFNS 2 Etot
PFDS 1 Etot
PFDS 2 Etot
PF11SA 1 Etot
PF11SA 2 Etot
PF12SA 1 Etot
PF12SA 2 Etot
1e6
Running Avg.
PFNS
PFDS
PF11SA
PF12SA
Etot (kcal/mol)
Time (ns)

Table S6 . Average per-residue decomposition energies for each PFAS simulation of non-polar pocket residues along with ITY residues.

PDB	PFBA	PFPA	PFOA	PFNA	PFDA	PFUnDA	PFDoDA	PFTrDA	PFPrS	PFBS	PFHpS	PFOS	PFNS	PFDS	PF11SA	PF12SA
**P677**	-0.99	-3.53	-2.35	-6.45	-3.80	-3.84	-5.89	-2.89	-2.01	-4.38	-5.37	-3.45	-4.04	-4.49	-3.90	-6.12
**A678**	0.98	1.97	1.59	3.86	2.06	1.15	2.80	1.42	1.81	1.63	1.98	0.48	1.03	1.89	1.53	3.89
**I2517**	0.26	-0.32	-0.45	-0.44	-0.18	0.00	-0.17	0.03	0.14	-0.18	-0.01	-0.03	-0.31	-0.22	-0.20	-0.47
**A2538**	-0.60	-0.77	-0.18	-0.24	-0.72	-0.51	-0.56	-0.41	-0.22	-0.38	-0.61	-0.67	-0.17	-0.54	-0.55	-0.52
**F2539**	-0.89	-1.18	-0.66	-1.18	-1.24	-1.57	-1.35	-0.66	-0.62	-0.89	-1.30	-0.93	-0.77	-0.96	-1.00	-1.41
**ITY2540**	-1.94	-1.00	-4.33	-11.25	-4.44	-5.26	-12.78	-2.70	-1.84	-2.39	-4.23	-3.29	-5.91	-2.99	-4.78	-11.40
**A2542**	-0.57	-0.64	-0.41	-0.76	-0.64	-0.85	-0.71	-0.40	-0.57	-0.53	-0.65	-0.55	-0.42	-0.51	-0.52	-0.82
**L2543**	-0.65	-0.82	-1.17	-1.60	-0.89	-1.29	-1.03	-0.56	-0.76	-0.80	-0.93	-0.74	-0.82	-0.80	-0.80	-1.43
**ITY2573**	-1.68	-8.67	-5.09	-8.93	-8.46	-10.96	-9.30	-8.82	-3.19	-11.16	-12.17	-5.95	-16.59	-12.68	-8.26	-10.26
**A2574**	-1.45	-1.45	0.10	-0.16	-1.16	-1.34	-1.51	-3.45	-1.02	-7.97	-2.24	-3.37	-9.42	-5.27	-1.41	0.49
**F2576**	-0.66	-1.57	-2.24	-4.16	-1.47	-1.47	-2.37	-1.03	-0.72	-2.37	-2.12	-1.32	-2.52	-2.03	-1.87	-3.71

Table S7. Average per-residue decomposition energies for each PFAS simulation of polar pocket residues.

PDB	PFBA	PFPA	PFOA	PFNA	PFDA	PFUnDA	PFDoDA	PFTrDA	PFPrS	PFBS	PFHpS	PFOS	PFNS	PFDS	PF11SA	PF12SA
**Q429**	0.23	-0.06	0.23	0.64	0.11	-1.91	1.63	1.55	0.24	-0.57	0.21	0.89	2.36	1.28	1.47	1.49
**S430**	-0.75	-0.81	-0.95	-0.69	-2.94	-1.27	-1.57	-3.72	-0.18	-11.48	-0.87	-1.61	-11.88	-2.10	-1.02	-1.48
**Q431**	-0.44	-1.84	-0.37	-0.75	-3.72	-0.39	-4.10	-1.43	-0.53	-4.63	-0.68	-6.29	-12.75	-6.59	-2.16	-0.51
**Q432**	0.07	-3.62	-1.39	-0.91	-1.43	-1.03	-0.34	-3.38	-0.43	-7.32	-1.44	-7.26	-6.11	-6.04	0.07	-0.26
**S675**	0.84	2.19	0.50	1.00	1.24	-0.29	2.36	1.54	0.99	3.61	2.45	2.08	2.47	2.68	2.93	1.02
**Q676**	-0.78	-1.15	-0.40	-0.51	-1.30	-0.39	-1.21	-4.47	-1.07	-0.18	-0.16	-5.36	-2.06	-1.84	-4.36	-1.42
**S2529**	-1.37	-0.24	-0.26	-0.58	-0.63	-0.31	-0.54	0.20	-0.62	-0.25	-0.19	-0.32	-0.14	-0.34	-0.38	-0.65
**T2537**	-1.37	-2.77	-2.42	-1.29	-2.24	0.02	-0.92	-2.04	-1.59	-1.07	-1.77	-2.28	-1.17	-2.08	-2.91	-2.19
**Q2541**	-1.08	-0.46	-3.37	-2.59	-0.28	-0.51	-0.25	-0.65	-0.17	0.04	-0.46	-0.89	-0.45	-0.24	-0.46	-2.53
**Q2544**	-0.05	-0.10	-2.15	-0.50	-0.11	-0.19	-0.17	0.11	0.04	-0.54	0.03	0.15	-0.16	0.29	0.17	-1.72
**N2545**	-0.73	-0.57	-1.38	-0.88	-0.43	-0.79	-0.40	-0.28	-0.54	-0.45	-0.55	-0.55	-0.29	-0.37	-0.39	-0.66
**S2569**	0.65	1.84	-3.92	-0.23	1.23	1.29	0.73	-0.24	0.54	0.04	1.83	-0.85	0.73	-1.12	0.25	0.17
**T2570**	0.59	0.46	-0.22	-0.91	0.18	0.35	0.15	-0.08	0.44	-0.23	0.17	-0.26	-0.03	-0.63	-0.26	-0.17
**S2575**	-0.67	-1.00	-0.63	-0.46	-0.87	-0.78	-1.06	-0.89	-0.49	-2.45	-1.31	-0.97	-3.47	-1.74	-0.38	-0.25
**S2577**	-1.03	-1.58	-0.97	-4.83	-1.80	-1.71	-3.96	-1.25	-0.75	-3.10	-2.64	-1.65	-2.76	-2.74	-3.59	-8.96

Table S8. Average per-residue decomposition energies for each PFAS simulation of basic pocket residues.

PDB	PFBA	PFPA	PFOA	PFNA	PFDA	PFUnDA	PFDoDA	PFTrDA	PFPrS	PFBS	PFHpS	PFOS	PFNS	PFDS	PF11SA	PF12SA
**R2530**	-46.14	-18.12	-17.90	-14.36	-15.07	-17.41	-16.65	-15.94	-17.38	-14.43	-16.78	-15.12	-18.06	-15.72	-16.93	-16.82
**K2534**	-26.36	-25.99	-30.52	-48.58	-35.14	-30.31	-49.27	-20.12	-41.65	-31.29	-45.98	-26.50	-25.59	-31.41	-32.77	-36.70
**K2536**	-44.72	-64.53	-45.77	-57.27	-64.72	-58.71	-75.28	-33.87	-38.89	-43.09	-58.03	-44.36	-33.62	-54.14	-49.82	-59.24
**R2578**	-17.71	-25.04	-22.09	-30.16	-26.20	-22.38	-30.77	-33.34	-23.77	-36.52	-31.41	-29.83	-30.06	-32.78	-31.91	-29.29

Table S9. Average per-residue decomposition energies for each PFAS simulation of acidic pocket residues.

PDB	PFBA	PFPA	PFOA	PFNA	PFDA	PFUnDA	PFDoDA	PFTrDA	PFPrS	PFBS	PFHpS	PFOS	PFNS	PFDS	PF11SA	PF12SA
**E2528**	30.67	35.79	27.23	32.37	38.26	35.05	41.96	31.23	29.90	27.40	41.10	30.01	26.09	30.73	30.84	34.27
**D2571**	19.41	25.05	21.93	22.71	22.41	22.61	20.36	24.58	16.61	33.53	24.00	26.26	32.29	31.01	20.79	21.96
**D2572**	19.71	27.24	24.24	27.77	30.33	24.78	28.56	27.81	24.89	37.32	38.18	29.80	41.30	32.12	29.30	29.67
**E2581**	15.70	18.60	19.82	23.78	18.86	18.19	21.78	18.47	21.66	19.48	20.15	18.62	18.93	19.76	18.98	23.40

Table S10. Hydrogen bond percentages of PFAS head group oxygen atoms. Res. ID: Residue ID of the amino acids.

	**Res. ID**	**PFBA**	**PFPrS**	**PFPA**	**PFBS**	**PFOA**	**PFHpS**	**PFNA**	**PFOS**	**PFDA**	**PFNS**	**PFUnDA**	**PFDS**	**PFDoDA**	**PF11SA**	**PFTrDA**	**PF12SA**
**PFAS@O**	**S430**	---	---	---	0.46	---	---	---	---	---	0.20	---	0.13	---	---	---	---
	**Q431**	---	---	---	0.31	---	---	0.10	---	---	0.55	---	0.23	---	---	---	---
	**T681**	---	0.14	---	---	---	---	---	---	---	---	---	---	---	---	---	---
	**K2524**	---	---	---	---	---	0.05	---	---	---	---	---	---	---	---	---	---
	**R2530**	0.5	---	---	---	---	0.05	---	---	---	---	---	---	---	---	---	---
	**K2536**	---	0.07	---	---	0.13	---	0.28	---	0.10	---	---	0.21	---	---	---	---
	**ITY2540**	---	---	---	---	---	---	---	---	---	---	---	---	0.43	---	---	---
	**S2569**	---	---	---	---	0.14	---	---	---	---	---	---	---	---	---	---	---
	**ITY2573**	---	---	---	0.74	---	---	---	0.12	---	0.24	---	0.37	---	---	---	---
	**A2574**	---	---	---	0.24	---	---	---	---	---	---	---	---	---	---	---	---
	**A2575**	---	---	---	---	---	---	---	---	---	---	---	---	---	---	---	0.40
**PFAS@O1**	**K2536**	---	---	0.12	---	---	---	0.08	---	0.19	---	---	---	0.40	---	---	---
	**A2574**	---	---	---	---	---	---	---	---	---	---	---	0.15	---	---	---	---
**PFAS@O2**	**A2572**	---	---	---	---	---	---	---	---	---	0.44	---	---	---	---	---	---

Table S11. Average hydrogen bond % fractions of Region 1. From left to right, the fluorinated carbon chain length increases.

	Residue Pair	APO	PFBA	PFPrS	PFPA	PFBS	PFOA	PFHpS	PFNA	PFOS	PFDA	PFNS	PFUnDA	PFDS	PFDoDA	PF11SA	PFTrDA	PF12SA
Region 1	Q676/R2578	0.75	0.66	0.84	0.21	0.69	0.42	0.40	0.71	0.82	0.63	0.67	0.38	0.63	0.65	0.66	0.70	0.13
	A678/S2577	0.42	0.00	0.17	0.59	0.56	0.55	0.00	0.51	0.54	0.55	0.44	0.40	0.56	0.54	0.61	0.45	0.37
	Q676/S680	0.00	0.00	0.11	0.06	0.32	0.16	0.21	0.00	0.34	0.16	0.28	0.07	0.29	0.57	0.39	0.24	0.00
	P677/S680	0.00	0.41	0.16	0.08	0.00	0.10	0.50	0.33	0.77	0.00	0.34	0.12	0.29	0.00	0.07	0.00	0.11
	G679/K2524	0.25	0.00	0.00	0.00	0.00	0.00	0.00	0.00	0.06	0.00	0.00	0.38	0.14	0.00	0.00	0.24	0.30
	G674/R2578	0.00	0.00	0.00	0.22	0.00	0.00	0.00	0.00	0.00	0.00	0.00	0.23	0.00	0.31	0.42	0.00	0.00
	A678/K2524	0.26	0.00	0.00	0.00	0.00	0.00	0.30	0.00	0.00	0.24	0.00	0.00	0.00	0.24	0.00	0.00	0.00
	S680/K2524	0.28	0.14	0.00	0.00	0.00	0.00	0.00	0.00	0.20	0.11	0.34	0.00	0.00	0.00	0.00	0.00	0.23
	Q676/T681	0.00	0.10	0.06	0.00	0.00	0.00	0.25	0.00	0.00	0.00	0.00	0.00	0.00	0.00	0.00	0.00	0.00
	S680/T681	0.00	0.09	0.00	0.00	0.00	0.00	0.00	0.00	0.00	0.00	0.00	0.00	0.00	0.00	0.00	0.00	0.00
	T681/L682	0.17	0.00	0.00	0.00	0.00	0.00	0.00	0.00	0.00	0.00	0.00	0.00	0.00	0.00	0.00	0.00	0.00
	P677/T681	0.09	0.00	0.00	0.00	0.00	0.00	0.00	0.00	0.00	0.00	0.00	0.00	0.00	0.00	0.00	0.00	0.00

Table S12. Average hydrogen bond %100 fractions of Region 2. From left to right, the fluorinated carbon chain length increases.

	Residue Pair	APO	PFBA	PFPrS	PFPA	PFBS	PFOA	PFHpS	PFNA	PFOS	PFDA	PFNS	PFUnDA	PFDS	PFDoDA	PF11SA	PFTrDA	PF12SA
Region 2	T2537/Q2541	0.35	0.27	0.24	0.19	0.37	0.17	0.34	0.37	0.42	0.15	0.36	0.43	0.38	0.13	0.33	0.34	0.45
	S2535/P2539	0.33	0.10	0.31	0.24	0.24	0.33	0.12	0.50	0.40	0.64	0.36	0.41	0.43	0.43	0.32	0.29	0.33
	ITY2540/K2536	0.25	0.19	0.29	0.29	0.00	0.06	0.03	0.36	0.41	0.57	0.51	0.54	0.36	0.46	0.39	0.52	0.33
	S2534/A2538	0.48	0.37	0.37	0.29	0.35	0.00	0.44	0.00	0.06	0.00	0.28	0.71	0.25	0.10	0.31	0.32	0.18
	G2524/T2533	0.00	0.00	0.11	0.00	0.12	0.22	0.00	0.00	0.06	0.20	0.00	0.29	0.30	0.18	0.33	0.12	0.05
	T2533/T2537	0.27	0.56	1.39	0.12	0.00	0.48	0.00	0.00	0.00	0.00	0.86	0.45	0.00	0.21	0.70	0.75	0.33
	ITY2540/A678	0.00	0.00	0.00	0.62	0.71	0.61	0.00	0.00	0.05	0.54	0.00	0.00	0.00	0.19	0.31	0.37	0.46
	S2534/T2537	0.00	0.00	0.00	0.22	0.33	0.00	0.00	0.00	0.05	0.27	0.00	0.17	0.48	0.00	0.00	0.00	0.23
	R2532/K2536	0.00	0.00	0.17	0.00	0.00	0.00	0.00	0.00	0.00	0.00	0.31	0.00	0.00	0.00	0.22	0.00	0.00
	R2532/S2535	0.00	0.00	0.00	0.00	0.00	0.00	0.00	0.00	0.00	0.00	0.00	0.63	0.12	0.57	0.51	0.10	0.26

Table S13. Average hydrogen bond 100% fractions of Region 3. From left to right, the fluorinated carbon chain length increases.

	Residue Pair	APO	PFBA	PFPrS	PFPA	PFBS	PFOA	PFHpS	PFNA	PFOS	PFDA	PFNS	PFUnDA	PFDS	PFDoDA	PF11SA	PFTrDA	PF12SA
**Region 3**	**ITY2573/S2577**	0.45	0.40	0.60	0.70	0.84	0.70	1.29	0.68	0.34	0.17	0.56	0.77	0.57	0.45	0.42	0.46	0.79
	**H2568/D2571**	0.44	0.53	0.31	0.36	0.14	0.75	0.81	0.21	0.00	0.53	0.28	0.28	0.00	0.24	0.95	0.00	0.43
	**H2568/D2572**	0.25	0.68	0.32	0.38	0.33	0.23	0.40	0.19	0.00	0.24	0.73	0.19	0.26	0.29	0.55	0.00	0.18
	**A2574/S2577**	0.44	0.70	0.00	0.64	0.56	0.81	0.00	0.44	0.85	0.00	0.34	0.82	0.40	0.35	0.44	0.52	0.29
	**T2570/D2571**	0.33	0.00	0.03	0.12	0.53	0.30	0.00	0.00	0.36	0.28	0.00	0.09	0.39	0.22	0.38	0.67	0.05
	**A2574/R2578**	0.21	0.14	0.58	0.10	0.00	0.30	0.22	0.08	0.00	0.28	0.00	0.08	0.22	0.06	0.47	0.25	0.00
	**D2572/S2575**	0.33	0.67	0.99	0.28	0.00	0.77	1.14	0.89	0.00	0.95	0.00	0.46	0.00	1.60	0.23	0.53	0.90
	**D2572/P2576**	0.39	0.00	0.07	0.47	0.00	0.10	0.00	0.00	0.00	0.00	0.00	0.41	0.24	0.00	0.19	0.40	0.00
	**D2572/A2574**	0.03	0.11	0.00	0.12	0.00	0.00	0.00	0.00	0.00	0.49	0.00	0.00	0.00	0.00	0.00	0.00	0.16
	**S2569/D2572**	0.00	0.00	0.00	0.00	0.00	0.00	0.00	0.00	0.84	0.00	0.00	0.00	0.00	0.00	0.00	0.25	0.00
	**ITY2573/Q2541**	0.00	0.00	0.11	0.00	0.00	0.00	0.00	0.00	0.00	0.00	0.00	0.00	0.00	0.00	0.00	0.00	0.00

Table S14. The MM-GBSA/PBSA binding energies of PFBA and PFPA with dimer hTG protein. The compounds did not form strong interactions with the binding site residues and did not reside within the region. These systems were simulated for 10 ns in two different poses, and their average is reported here.

**Simulation**	**MM-PBSA (kcal mol $^{-1}$)**		**MM-GBSA (kcal mol $^{-1}$)**	
	**dG**	**std**	**dG**	**std**
**PFBA (monomer hTG)**	-5.93	2.73	-5.30	1.86
**PFBA (monomer hTG)**	-3.69	2.95	-7.60	2.33
**PFBA (dimer hTG)**	10.80	5.67	-1.57	3.07
**PFPA (dimer hTG)**	-7.77	3.19	-9.02	2.31

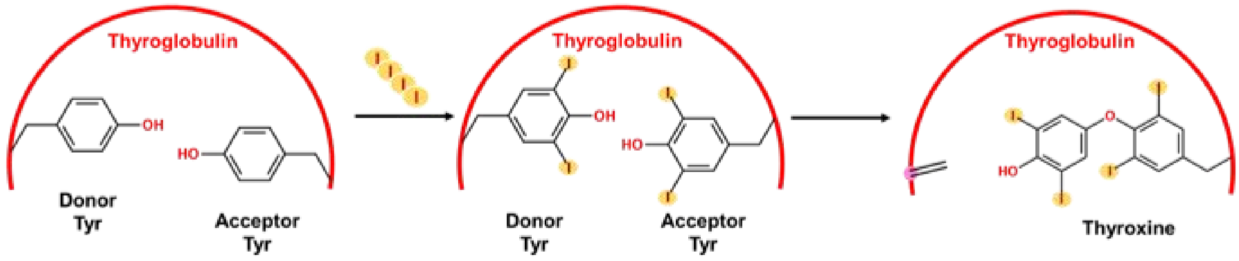

Figure S1. Formation of T4 by hormonogenic Tyrosine residues. Iodine is shown with yellow spheres.

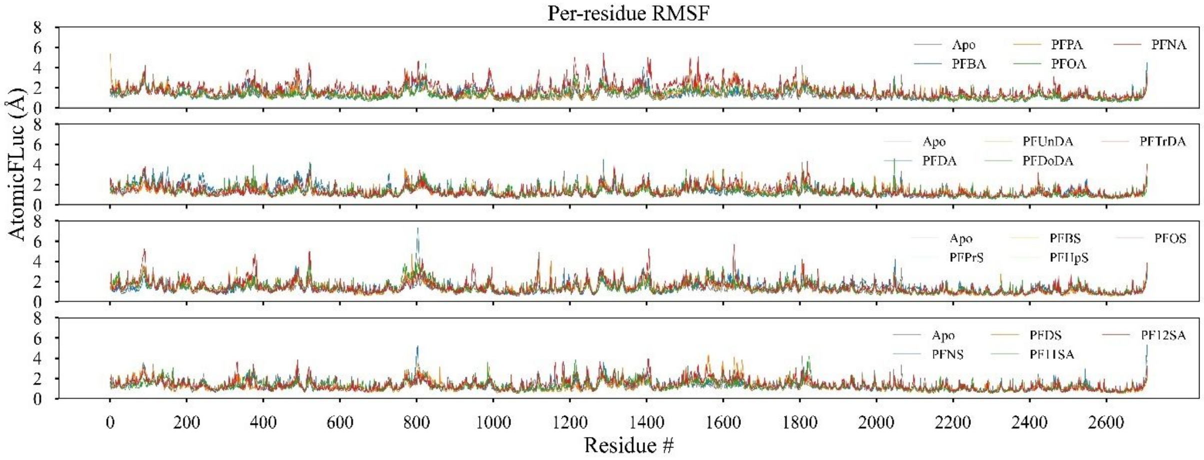

Figure S2. Per-residue RMSF plot of first simulation set.

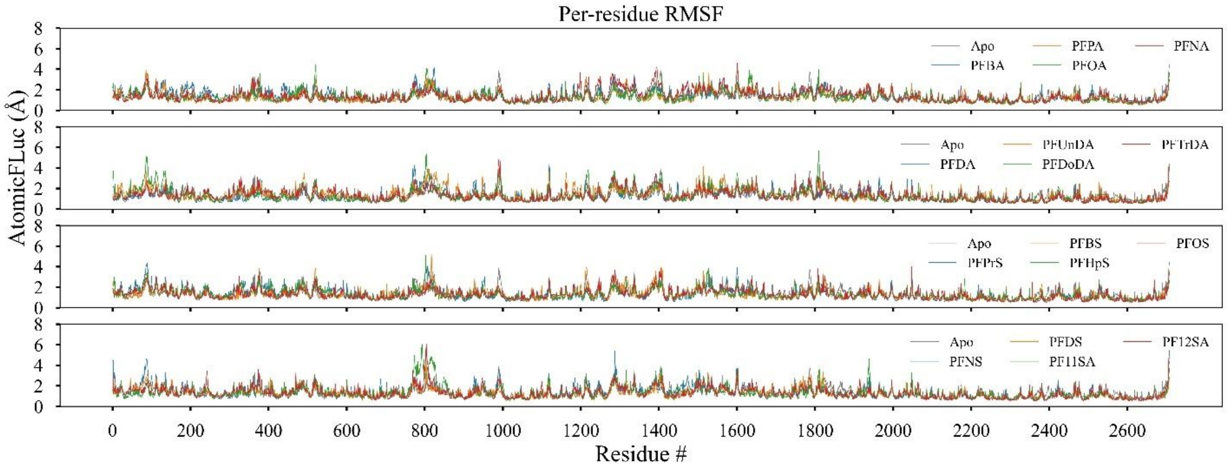

Figure S3. Per-residue RMSF plot of second simulation set.

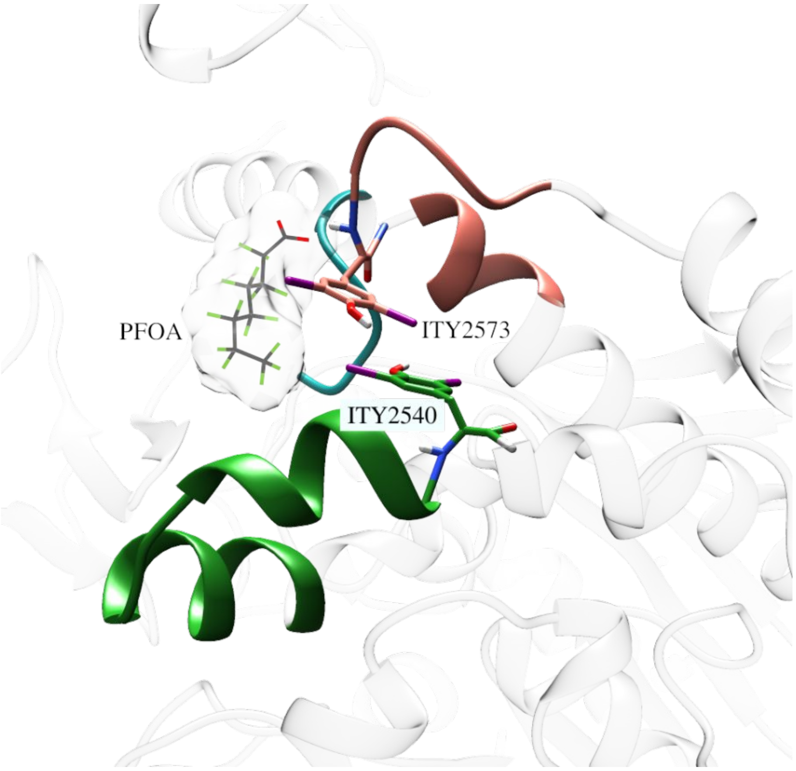

Figure S4. The regions for which the hydrogen bond patterns were investigated. Region 1 is shown in blue and includes S675, Q676, P677, A678, G679, and S680 residues. Region 2 is shown in green and includes V2523, K2524, Q2525, F2526, E2527, E2528, S2529, R2530, G2531, R2532, T2533, S2534, S2535, K2536, T2537, A2538, F2539, and ITY2540. Region 3 is depicted in pink loop representation and has the following residues: H2568, S2569, T2570, D2571, D2572, ITY2573, A2574, S2575, F2576, S2577, and R2578. The rest of the Thyroglobulin protein is shown as cartoon in grey color. PFOA is shown in wire representation, and ITY residues are shown in stick representation.

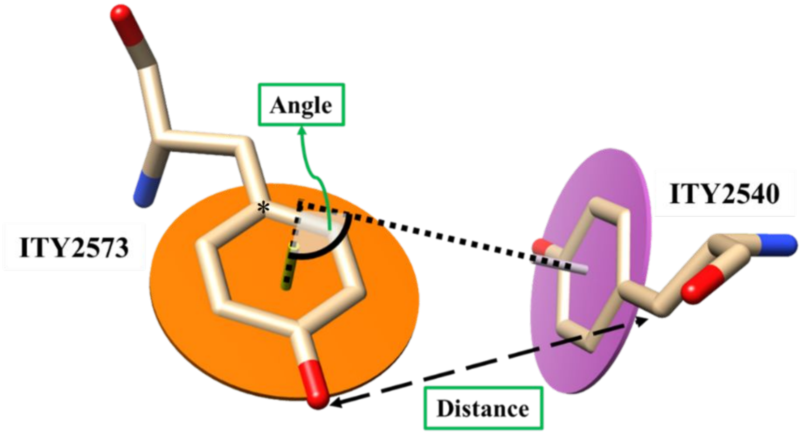

Figure S5. Representation of angle and distance measurements between ITY2540 and ITY2573. The residues are shown in stick representation, and the planes, shown as disks, are created by considering the side chain ring atoms. The normal of planes are shown as sticks. The distance between the OH atom of ITY2540 and CB atom of ITY2573 is shown with a dashed black arrow. CG atom is indicated by asterisk (*) on ITY2573, for reference. The structure of ITY residues is taken from the cryo-EM structure (PDB ID: 6SCJ). The distance calculated for the structure is 6.4 Å and the angle is 76 °.

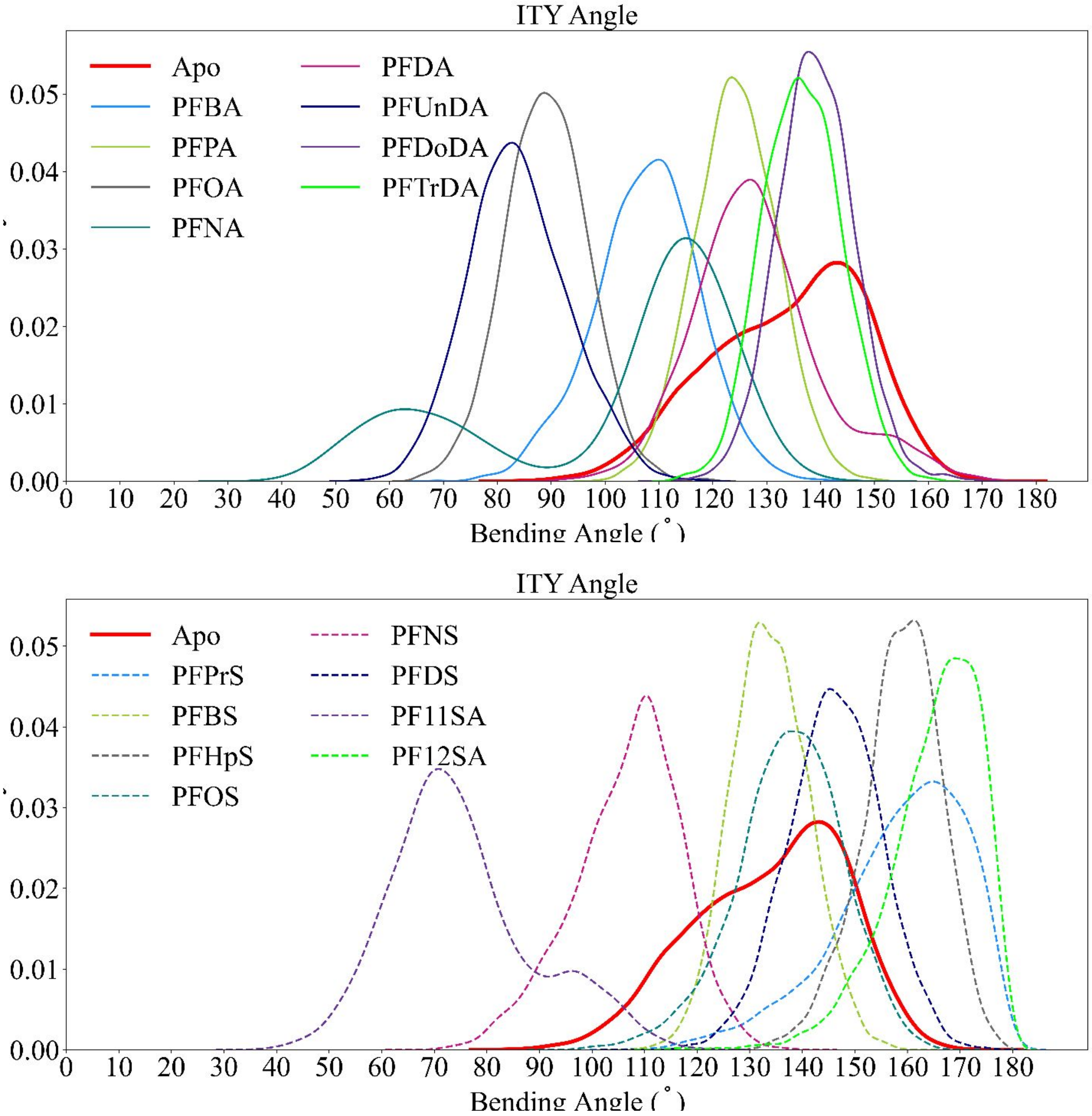

Figure S6. The kernel density plot of the distribution of calculated angles between the ITY residues. Above: PFCA compounds, below: PFSA compounds. Apo system is shown in solid red line in both plots.

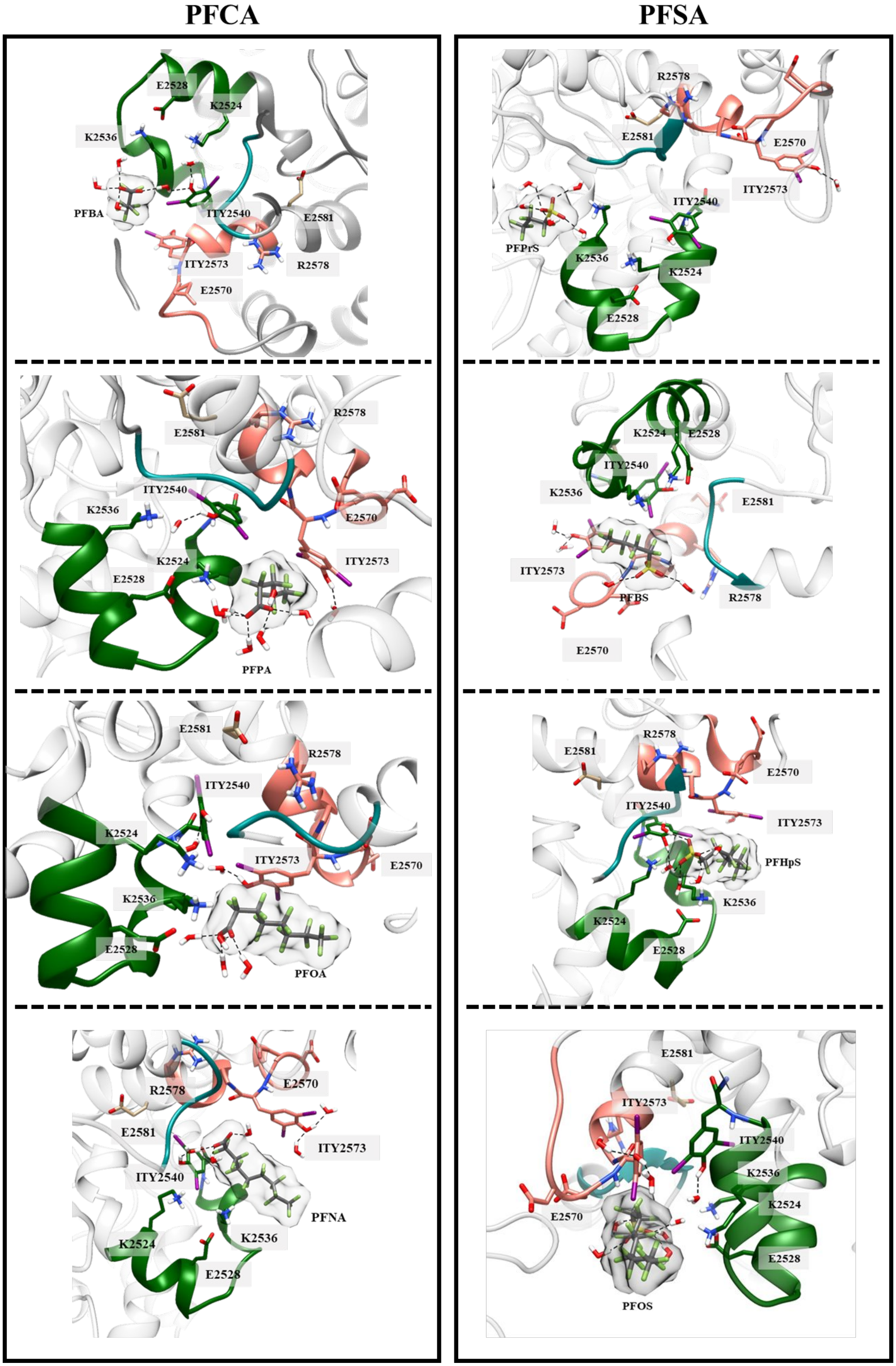
PFCA
PFSA
E2528
K2524
K2536
PFBA
ITY2540
E2581
ITY2573
R2578
E2570
R2578
E2581
E2570
ITY2573
ITY2540
PFPrS
K2536
K2524
E2528
R2578
E2581
ITY2540
K2536
E2570
K2524
ITY2573
E2528
PFPA
K2524
E2528
ITY2540
K2536
E2581
ITY2573
PFBS
R2578
E2570
E2581
R2578
ITY2540
K2524
ITY2573
E2570
K2536
E2528
PFOA
R2578
E2581
E2570
ITY2540
ITY2573
PFHpS
K2536
K2524
E2528
E2570
R2578
E2581
ITY2573
ITY2540
PFNA
K2524
K2536
E2528
E2581
ITY2573
ITY2540
K2536
E2570
K2524
E2528
PFOS

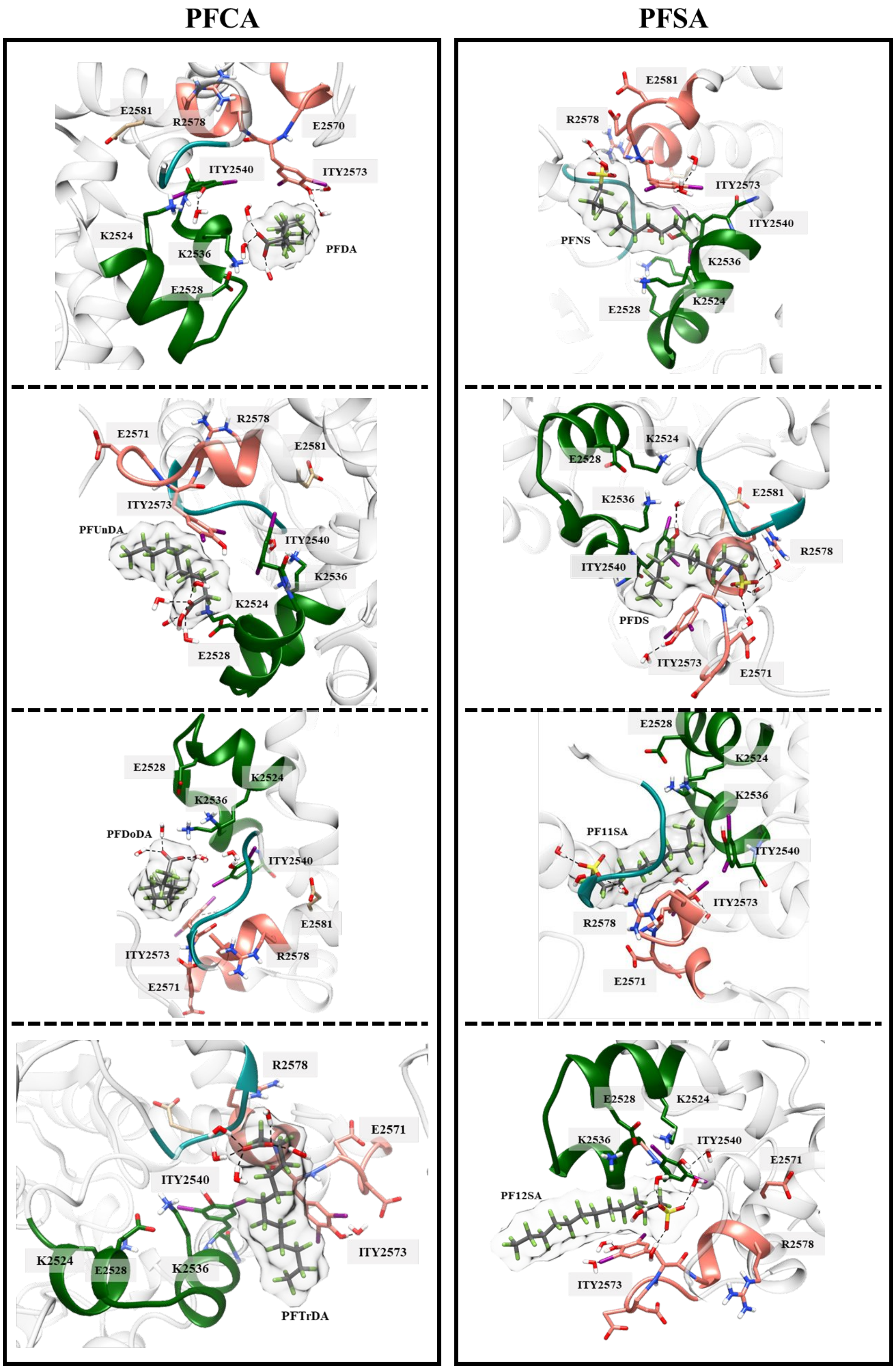
PFCA
PFSA
E2581
R2578
E2570
ITY2540
ITY2573
K2524
K2536
PFDA
E2528
E2581
R2578
ITY2573
ITY2540
PFNS
K2536
E2528
K2524
R2578
E2571
E2581
ITY2573
PFUnDA
ITY2540
K2536
K2524
E2528
K2524
E2528
K2536
E2581
R2578
ITY2540
PFDS
ITY2573
E2571
E2528
K2524
K2536
PFDoDA
ITY2540
E2581
ITY2573
R2578
E2571
E2528
K2524
K2536
PF11SA
ITY2540
ITY2573
R2578
E2571
R2578
E2571
ITY2540
ITY2573
K2524
E2528
K2536
PFTrDA
E2528
K2524
K2536
ITY2540
E2571
PF12SA
R2578
ITY2573

Figure S7. The dominant orientations of ITY residues and PFAS compounds, extracted by clustering the last 5ns of the simulations. The key residues that have either the highest/lowest interaction with PFAS or that make hydrogen bonds with PFAS were shown in stick representation. The coloring of the secondary structures was based on the scheme shown in **Figure S4.** PFCA: per-fluoroalkyl carboxylic acid, PFSA: per-fluoroalkyl sulphonic acid. The figure is obtained using Chimera.

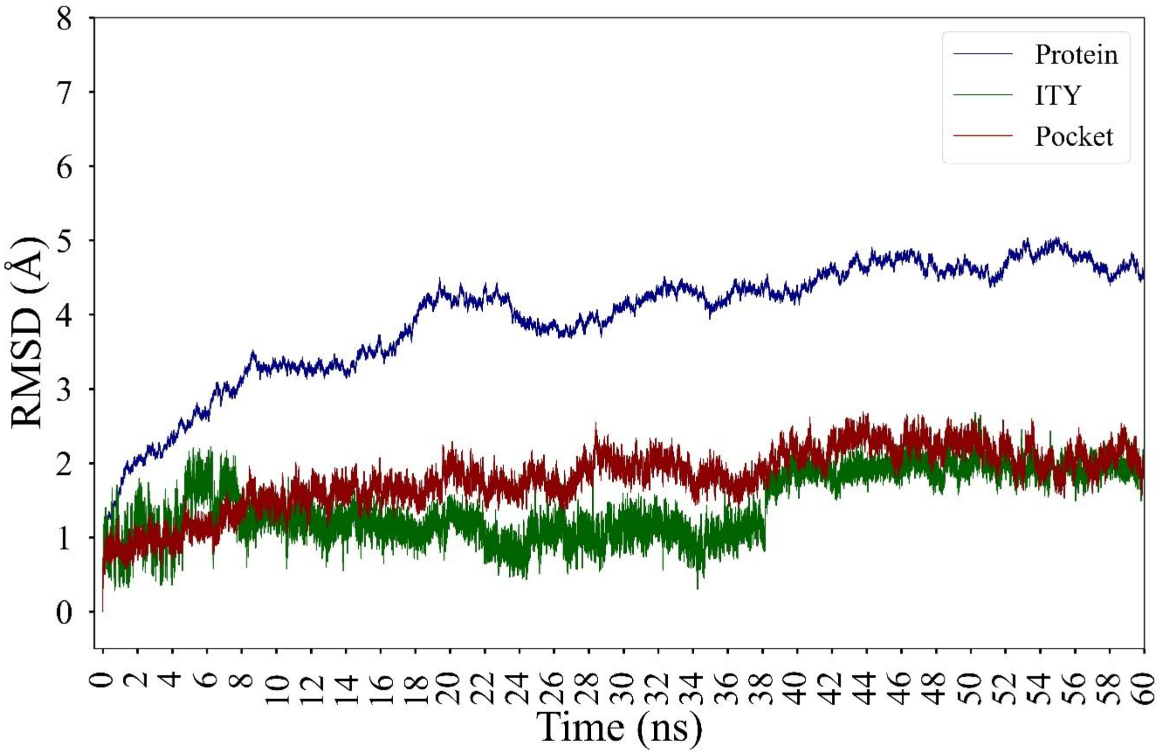

Figure S8. RMSD plot of apo hTG dimer protein is shown. The purple line represents the whole-protein backbone RMSD, and the green line represents the RMSD of the heavy atoms in the pocket residue.

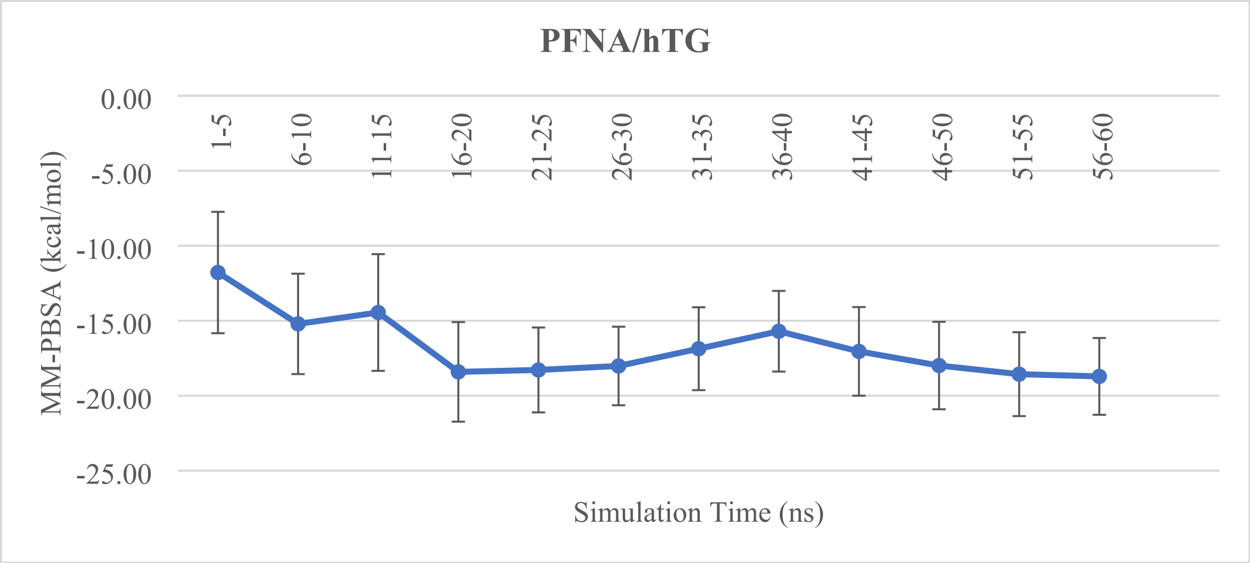

Figure S9. The MM-PBSA binding energies for PFNA compound. The simulations were performed for 60 ns in duplicate and the binding energies were calculated for every ns, then averaged for 5 ns windows. 16-20 ns range is the value reported in the **Figure 1(c)**.

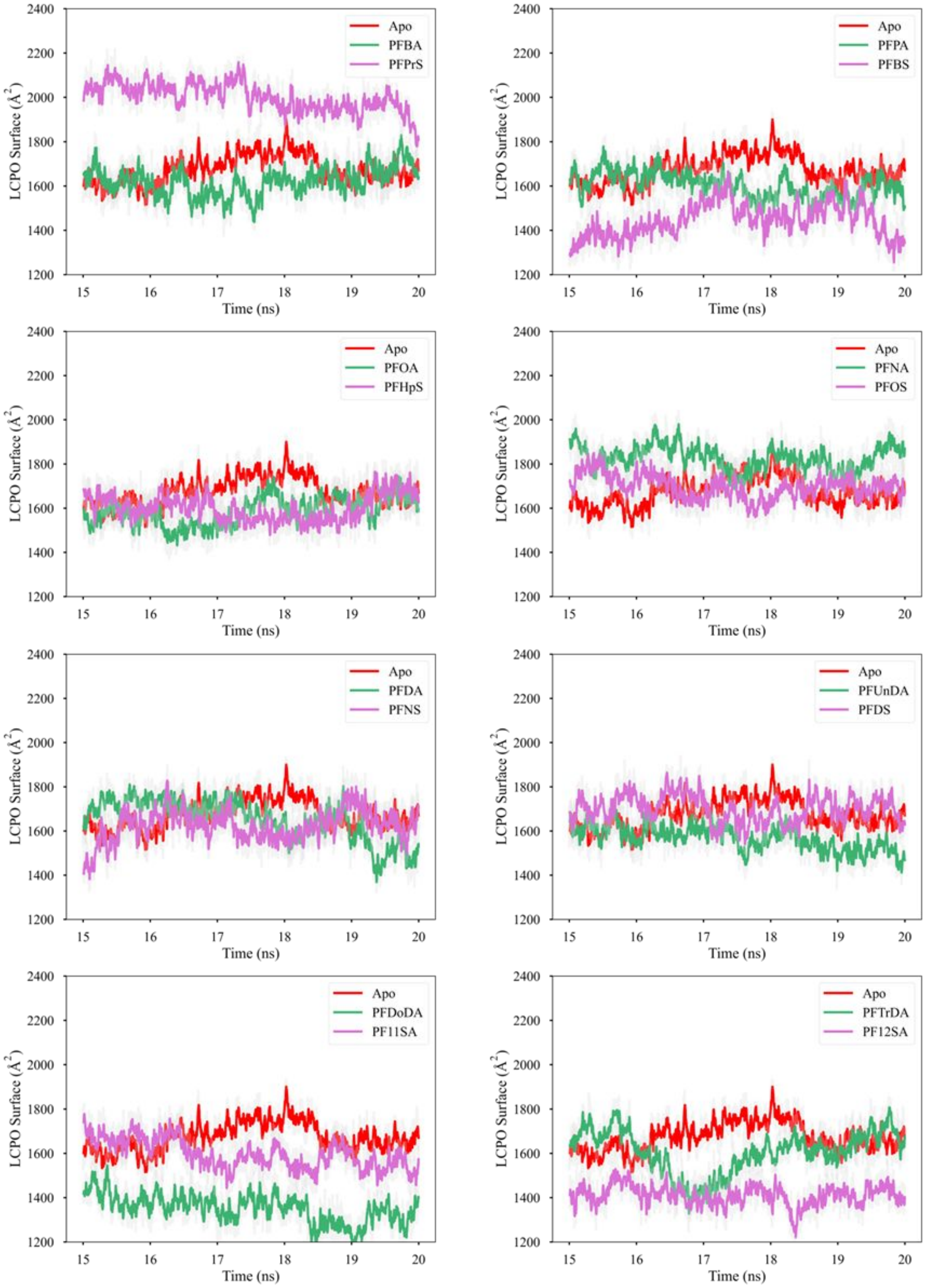

Figure S10. Surface Area calculations of ITY residues in the presence of PFAS.
